# Perinatal and Early-Life Nutrition, Epigenetics, and Allergy

**DOI:** 10.3390/nu13030724

**Published:** 2021-02-25

**Authors:** Nathalie Acevedo, Bilal Alashkar Alhamwe, Luis Caraballo, Mei Ding, Antonio Ferrante, Holger Garn, Johan Garssen, Charles S. Hii, James Irvine, Kevin Llinás-Caballero, Juan Felipe López, Sarah Miethe, Khalida Perveen, Elke Pogge von Strandmann, Milena Sokolowska, Daniel P. Potaczek, Betty C. A. M. van Esch

**Affiliations:** 1 Institute for Immunological Research, University of Cartagena, Cartagena 130014, Colombia; nacevedoc@unicartagena.edu.co (N.A.); lcaraballog@unicartagena.edu.co (L.C.); llinaskevin@gmail.com (K.L.-C.); jflocre@hotmail.com (J.F.L.); 2 Institute of Tumor Immunology, Clinic for Hematology, Oncology and Immunology, Center for Tumor Biology and Immunology, Philipps University Marburg, 35043 Marburg, Germany; bilal.alashkaralhamwe@staff.uni-marburg.de (B.A.A.); poggevon@staff.uni-marburg.de (E.P.v.S.); 3 College of Pharmacy, International University for Science and Technology (IUST), Daraa 15, Syria; 4 Swiss Institute of Allergy and Asthma Research (SIAF), University of Zurich, 7265 Davos, Switzerland; mei.ding@siaf.uzh.ch (M.D.); milena.sokolowska@siaf.uzh.ch (M.S.); 5 Christine Kühne-Center for Allergy Research and Education, 7265 Davos, Switzerland; 6 Department of Allergology, Zhongnan Hospital of Wuhan University, Wuhan 430071, China; 7 Department of Immunopathology, SA Pathology at the Women’s and Children’s Hospital, North Adelaide, SA 5006, Australia; Antonio.Ferrante@sa.gov.au (A.F.); charles.hii@adelaide.edu.au (C.S.H.); james@hearingmatters.com.au (J.I.); khalida.perveen@adelaide.edu.au (K.P.); 8 Adelaide School of Medicine and the Robinson Research Institute, University of Adelaide, Adelaide, SA 5005, Australia; 9 School of Biological Sciences, University of Adelaide, Adelaide, SA 5005, Australia; 10 Translational Inflammation Research Division & Core Facility for Single Cell Multiomics, Medical Faculty, Philipps University Marburg, Member of the German Center for Lung Research (DZL) and the Universities of Giessen and Marburg Lung Center, 35043 Marburg, Germany; garn@staff.uni-marburg.de (H.G.); miethes@staff.uni-marburg.de (S.M.); 11 Division of Pharmacology, Utrecht Institute for Pharmaceutical Sciences, Faculty of Science, Utrecht University, 3584 CG Utrecht, The Netherlands; j.garssen@uu.nl; 12 Danone Nutricia Research, 3584 CT Utrecht, The Netherlands

**Keywords:** allergic disease, asthma, breastfeeding, environmental factors, epigenetic mechanisms, DNA methylation, histone modifications, metabolic programming, microbiome, microRNA (miRNA), milk, neonatal T cells, nutritional interventions, perinatal, polyunsaturated fatty acids (PUFA), vitamins

## Abstract

Epidemiological studies have shown a dramatic increase in the incidence and the prevalence of allergic diseases over the last several decades. Environmental triggers including risk factors (e.g., pollution), the loss of rural living conditions (e.g., farming conditions), and nutritional status (e.g., maternal, breastfeeding) are considered major contributors to this increase. The influences of these environmental factors are thought to be mediated by epigenetic mechanisms which are heritable, reversible, and biologically relevant biochemical modifications of the chromatin carrying the genetic information without changing the nucleotide sequence of the genome. An important feature characterizing epigenetically-mediated processes is the existence of a time frame where the induced effects are the strongest and therefore most crucial. This period between conception, pregnancy, and the first years of life (e.g., first 1000 days) is considered the optimal time for environmental factors, such as nutrition, to exert their beneficial epigenetic effects. In the current review, we discussed the impact of the exposure to bacteria, viruses, parasites, fungal components, microbiome metabolites, and specific nutritional components (e.g., polyunsaturated fatty acids (PUFA), vitamins, plant- and animal-derived microRNAs, breast milk) on the epigenetic patterns related to allergic manifestations. We gave insight into the epigenetic signature of bioactive milk components and the effects of specific nutrition on neonatal T cell development. Several lines of evidence suggest that atypical metabolic reprogramming induced by extrinsic factors such as allergens, viruses, pollutants, diet, or microbiome might drive cellular metabolic dysfunctions and defective immune responses in allergic disease. Therefore, we described the current knowledge on the relationship between immunometabolism and allergy mediated by epigenetic mechanisms. The knowledge as presented will give insight into epigenetic changes and the potential of maternal and post-natal nutrition on the development of allergic disease.

## 1. Introduction

### 1.1. Allergic Diseases

Allergic diseases continue to pose a major burden to the health-care system. They are caused by hypersensitivity, an undesirable and inappropriate response of the immune system to otherwise harmless environmental substances called allergens. Based on the underlying immunological mechanism, allergic diseases can be roughly classified as either those that are immunoglobulin E (IgE)-mediated or -independent. The first group comprises atopic asthma, allergic rhinitis (AR), allergic conjunctivitis, IgE-dependent form of atopic dermatitis (AD), and food allergy. Non-atopic asthma, contact dermatitis, non-IgE-dependent form of AD, and non-IgE-mediated food allergy belong to the second group [1,2,3,4,5,6,7].

Despite substantial pathobiological differences between specific allergic disorders and among their heterogeneous phenotypes, some core mechanisms are known. This is true especially in the case of IgE-dependent entities underlain in general by chronic allergic inflammation with recurring acute episodes. The repertoire of cellular contributors is wide, with major players comprising epithelial cells, antigen-presenting cells (APCs), T and B cells, mast cells, as well as basophils, eosinophils and neutrophils. The role of epithelium extends far beyond a simple mechanical barrier protecting the tissue from negative environmental influences and also includes the integration of innate and adaptive immune mechanisms. Mediators secreted by the epithelium triggered by allergens participate in the stimulation of APCs which activate allergen-specific T cells [1,4,8,9]. T cells play various roles including effector functions, orchestration of adaptive immune responses and activation of B cells to differentiate to plasma cells to produce allergen-specific IgE. [2,10,11]. Some allergy-related effects typically associated with T cells can also be exerted by innate lymphoid cells (ILCs) [8]. IgE molecules occupy their high-affinity receptors on the surface of basophils and mast cells. By cross-linking those IgE/high-affinity IgE receptor (FcεRI) complexes, allergens stimulate the release of local bioactive mediators such as histamine from the cells, which ultimately leads to immediate allergic responses and allergic inflammation [12,13,14].

### 1.2. Epigenetic Mechanisms

Epigenetic biomarkers are mitotically and/or meiotically heritable but reversible, functional, and biologically relevant biochemical modifications of the chromatin carrying the information but not changing the nucleotide sequence of the genome [15,16,17,18,19]. DNA methylation and histone modifications represent “classical” epigenetic mechanisms [9,20]. Classical epigenetic modifications are best known for their effects on the accessibility of genes to transcriptional machinery and thus regulation of gene expression, occurring during activation or differentiation programs, or in the frames of cellular hemostasis or in response to environmental influences or as an important element of cellular homeostasis, and their contribution to the response to DNA damage [2,21].

**Figure 1:**
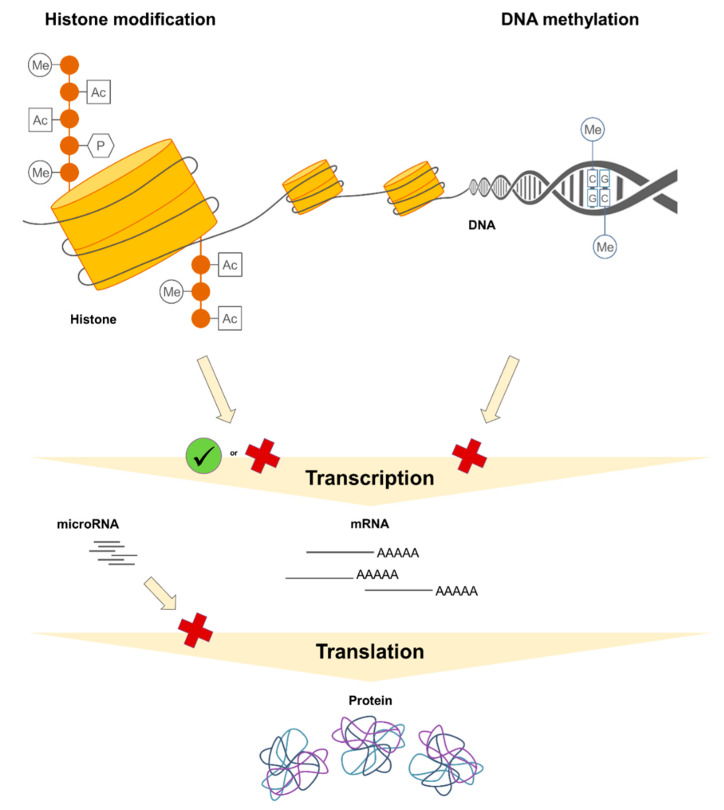
Molecular basics of the epigenetic mechanisms and their role in the expression control. For details, please refer to the main text, Chapter 1.2. *Epigenetic mechanisms*. Me, methylation; Ac, acetylation; P, phosphorylation; mRNA, messenger RNA.

From a biochemical point of view, DNA methylation is an enzymatically catalyzed covalent transfer of a methyl group onto the cytosine, occurring typically at cytosine nucleotides within CpG dinucleotides, so-called “CpG sites”. CpG sites are DNA sequences in which a cytosine nucleotide (C) is directly followed by a guanine nucleotide (G) [22,23]. CpG sites may tend to cluster, thus forming so-called “CpG islands”, which usually locate in the elements regulating gene transcription such as promoters or enhancers [24]. Low DNA methylation levels in promoter regions frequently, although not always, associate with a higher transcriptional activity, while high promoter DNA methylation levels are often related to lower gene expression or even full gene silencing (Figure 1) [25,26].

Histone modifications, including acetylation, methylation, phosphorylation, sumoylation, ubiquitination, and others, are best known for their role in the regulation of chromatin structure and therefor transcriptional activity and gene expression. In addition, they are also involved in other biological processes. For instance, histone phosphorylation contributes to DNA repair processes in response to cell damage [27,28,29,30,31]. Biochemically, histone modifications are enzymatically catalyzed additions of the respective moieties to amino acid residues including lysines or arginines but also serines, threonines, tyrosines, and others [31,32]. Higher levels of histone acetylation are usually associated with increased transcriptional activity and thus gene expression. Depending on the number of methyl groups added and the position of the targeted amino acid residue in the histone tail, histone methylations may have a transcriptionally permissive or repressive character (Figure 1) [2,20,32,33,34,35].

Regulatory non-coding RNAs such as microRNAs (miRNA) can be considered as “non-classical” epigenetic mechanisms and contribute to the post-transcriptional control of gene expression [36,37,38,39]. miRNA molecules are about 22 nucleotides long. They are highly abundant in the genome, with more than 2500 mature human miRNAs identified so far. Mature miRNA molecule exerts its effects within the RNA-induced silencing complex (RISC), in which it is responsible for the specific recognition of and interaction with the respective target messenger RNA (mRNA). The effects of RISC on targeted mRNA molecules include mRNA degradation or translation suppression, for example through reduction in the ribosomal performance (Figure 1) [38,40,41].

### 1.3. Perinatal and Early-Life Period—Window of Susceptibility, Window of Opportunity

Epidemiological observations have shown a dramatic increase in the incidence and the prevalence of allergic diseases over last several decades that cannot be explained by genetic variations. Epidemiological studies have also shown that this increase occurred most probably due to environmental alterations including lower exposure to protective factors (e.g., farming) and higher exposure to risk factors (e.g., pollution). The influences of those changing environmental factors are thought to be mediated by epigenetic mechanisms interacting with a genetic background, which makes allergic diseases prototypic example disorders, mechanisms of which are based on gene–environment interplay [26,42,43,44,45].

Although it does not necessarily mean that environmental exposures are completely unable to affect biological features and thus disease predisposition outside of this time range, an important feature characterizing epigenetically-mediated influences of various environmental factors is the existence of a time window within which their effects are the strongest and therefore most crucial. This timeframe corresponds to pregnancy, the neonatal period, and the first year of life, and is the optimal time for protective factors to exert their beneficial effects (“window of opportunity”) and the risk factors to increase the chance of disease development (“window of vulnerability” or “susceptibility”) [2,46]. In utero and early childhood periods also constitute an oportunity for epigenetic intervention (“window of intervention”), for instance with the so-called “epigenetic diet” which might be capable of preventing or even reverting the negative effects of environmental risk factors [47,48].

## 2. The effects of Nutrients on Neonatal T Cell Development

### 2.1. T helper (Th) Cell Balance and Allergy

**Figure 2:**
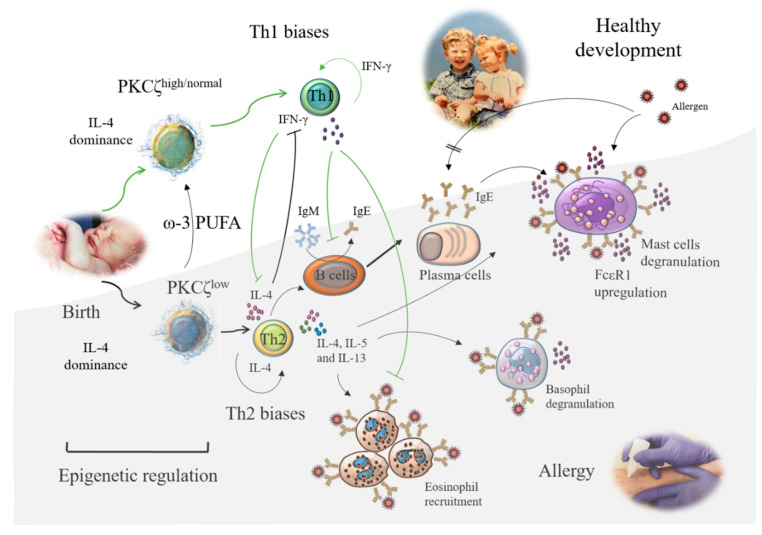
Schematic representation of the maturation of T cells perinatally, their regulation by protein kinase C ζ (PKCζ), and the influence of nutrients on predisposition to allergy. The immature T cells at birth can exhibit different levels of PKCζ, despite showing a dominance of interleukin 4 (IL-4) production over interferon γ (IFN-γ). Low levels promote skewing towards a type 2 T helper (Th2) cytokine profile and are associated with allergy development. Cytokines from the type 1 T helper (Th1) and Th2 cells influence the production of immunoglobulin E (IgE) and the effector cells, mast cells, basophils, and eosinophils of the allergic reaction. Omega-3 polyunsaturated fatty acid (ω3 PUFA) supplementation leads to upregulation of PKCζ expression via an epigenetic mechanism, rebalancing the skewed Th2 development and preventing allergy. The pictures shown in this diagram are from the annual reports of the Robinson Research Institute, University of Adelaide. IgM, immunoglobulin M; FcεR1, high-affinity IgE receptor.

The type 2 Th (Th2) dominance is a key feature of atopy or allergy as these cells predominantly produce interleukin 4 (IL-4), IL-5, and IL-13, which are involved in the initiation and perpetuation of the allergic phenotype. IL-4, which is produced predominantly by naïve CD4^+^ and CD8^+^ T cells in humans [49,50] and mice [51,52], promotes the differentiation of naïve T cells towards Th2, immunoglobulin class switch to IgE in B cells, and upregulation of FcεRI expression on mast cells. IL-5 is important in the activation, development, survival, and differentiation of eosinophils and mast cells and enhances degranulation of basophils (Figure 2). IL-13 is also responsible for immunoglobulin class switch to IgE, mast cell activation, and mucus production by epithelial cells, and promotes eosinophil trafficking to mucosal sites. On the other hand, type 1 Th (Th1) cytokine interferon γ (IFN-γ) is inhibitory toward the development of Th2 cells [53,54,55] (Figure 2). Thus, it is tempting to speculate that a well-balanced Th1 to Th2 cell immune response is desirable to achieve effective defense against infection and cancer but concomitantly reducing the risk of developing allergic and autoimmune inflammatory conditions. Indeed, it is not surprising that a substantial amount of effort is being made to re-balance the skewed Th2 immunity in allergic diseases (Figure 2).

Differentiation of Th1 and Th2 cells, as well as of the other Th cell populations playing a crucial role in allergies such as regulatory T cells (Treg cells), type 17 Th (Th17) cells, and type 9 Th (Th9) cells, is strictly controlled by epigenetic mechanisms [2,10,56]. Thus, in line with the statements in Chapter 1.3., the arising question is whether there is a “window” in the perinatal period not only to identify those at risk of developing allergic diseases (window of susceptibility), but also to then implement a nutritional exposure to prevent this pathway by influencing T cell epigenetics (window of opportunity). Furthermore, an involvement of type 2 innate lymphoid cells (ICL2s) which promote naïve T cells towards Th2 and suppressing Th1 development needs to be considered [57,58], although the numbers of ILC2s in cord blood (CB) did not correlate with the development of allergy [59].

### 2.2. Protein Kinase C (PKC) ζ (PKCζ) Promotes Neonatal T Cell Development towards a Th1 Anti-Allergy Phenotype

The perinatal period is characterized by an adaptive Th2 bias, reflecting the immunomodulatory milieu of pregnancy. Thereafter, normal immune maturation depends on effective switching to more mature and regulated Th1 responses. A continual propensity for a Th2 functional phenotype is associated with predisposition to allergic responses. Some insight into the molecular basis for this continued Th2 propensity has been gained. CB T cells (CBTCs) from a significant number of babies from women with a family history of allergy were found to express low PKCζ levels [60]. There was an inverse correlation between these levels in immature CBTCs and the development of allergic sensitization at the ages of 1 to 2.5 years. Using these data, it was possible to establish that CBTC PKCζ levels of <62.3% (relative to expression by T cells from blood of adults) would predict allergic disease development with an accuracy of 71%, sensitivity of 80% and specificity of 63%. This is a major development, as the epidemic rise in allergic diseases in the last four decades requires an urgent need to identify the critical pathway(s) that lead to disease. As we identify better intervention approaches, it will be crucial to diagnose those children with an allergy risk. Considering that there are no biological biomarkers that are considered of predictive value, PKCζ remains currently potentially a valuable protein marker. While IgE has been extensively evaluated as a predictive marker, this has been found to be unreliable [61]. Measuring cytokines at this stage of immune development has also resulted in a fruitless approach, leaving only the crude assessment of “family history” as the currently available predictor for allergy development.

It was also evident that low levels of PKCζ in the CBTC correlated with the development and maturation of cells with a propensity to display Th2 cytokine responses [62,63,64]. The data imply that the PKCζ level in immature T cells at birth may be a key determinant in allergy development in childhood and adulthood. These findings also argue that the intrinsic inability of immature neonatal T cells to produce a normal immune response is related to their PKCζ expression level in some neonates and this needs to be considered with findings that phospholipase (PLC) β2 (PLCβ2) and γ1 (PLCγ1), lymphocyte specific protein tyrosine kinase (LCK) proto-oncogene, Src family tyrosine kinase (p56lck), and ζ-chain of T cell receptor-associated protein kinase 70 (ZAP70) are also deficient [65]. Examination of the activation of the mitogen-activated protein (MAP) kinases 1 (ERK) and 8 (JNK) in CBTC showed that while activation of these via the non-PKC-mediated pathway was normal, they failed to be activated through the engagement of the T cell receptor (TCR) or PKC, using anti-CD3/anti-CD28 and phorbol 12-myristate 13-acetate (PMA) stimulation, respectively. This was not related to an inability to activate PKC but significantly involved low expression of some isozymes of PKC. While the levels of these PKC isozymes were not essential to the development of T cells from the immature CD45RA to the mature CD45RO phenotype, it was critical to whether the cells developed to Th1 (IFN-γ producing) or Th2 (IL-13 producing) cell types [62,63,64].

**Figure 3:**
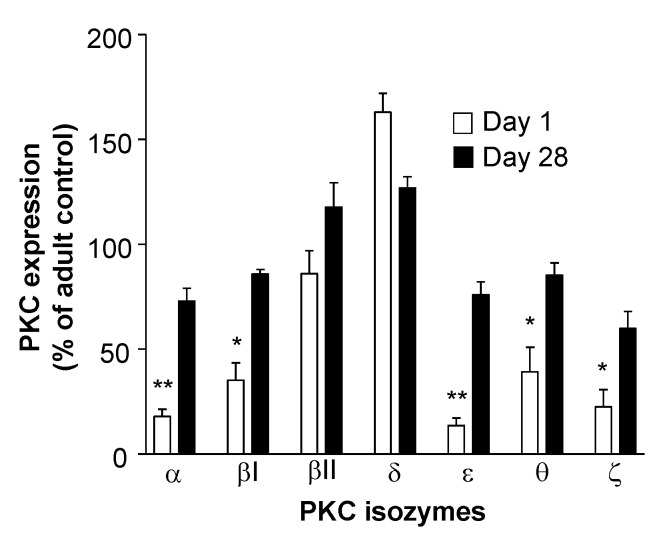
Protein kinase C (PKC) isoform expression in mouse neonates is upregulated in the first 4 weeks of life to near adult levels. Nylon wool column purified splenic T cells from 14 newborn Swiss white mice or 28-day-old mice were lysed and equivalent amounts of protein were resolved by western blot [66]. PKC isoform expression was visualized using isoform-specific polyclonal antibodies (PKC βI, βII, δ, ε, θ, and ζ) followed by densitometric band analysis using Image Quant software. Data are expressed as mean ± standard error of the mean of PKC isoform expression for newborn and day-28 mice relative to the PKC isozymes from T cells of adult mice. Statistics: *, *p* < 0.05; **, *p* < 0.01.

**Figure 4:**
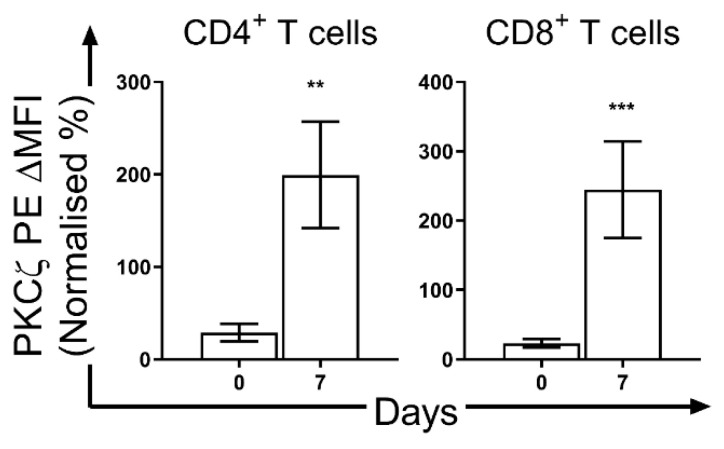
Changes in protein kinase C ζ (PKCζ) levels in human cord blood (CB) CD4^+^ and CD8^+^ T cells during in vitro maturation. T cells expressing low PKCζ levels were matured by culturing the CB mononuclear cells in the presence of phytohemagglutinin (PHA; 2 μg/mL) and interleukin 2 (10 ng/mL) [63]. Levels of PKCζ were measured by flow cytometry. Results are mean ± standard deviation of 4 experiments [66]. Statistics: **, *p* < 0.01; ***, *p* < 0.001. PE, phycoerythrin; MFI, mean fluorescence intensity, expressed as a % of standard value of cryopreserved T cells from adults measured in the same experimental run.

The low expression of these PKC isozymes including PKCζ is a transient state which subsequently increases to levels seen in adult T cells towards the end of the neonatal period. However, as no studies have been undertaken to examine T cell PKCζ levels during neonatal development in vivo, a significant increase in PKCζ by the end of the neonatal period was found in mice (Figure 3). In the CBTC maturation culture model, it has been observed that this low PKCζ state at birth is transient and when the T cells have matured and express the CD45RO^+^/RA^-^ phenotype on day 7, the levels are normal (Figure 4) [66]. This normalization of PKC levels is important for the mature T cells to respond effectively through the TCR. However, of importance is whether this development can be reprogrammed by the nature of the environmental/nutritional exposure during the prenatal period.

### 2.3. Nutritional Factors May Affect PKCζ-Mediated Th1 Bias through Epigenetic Mechanisms

Maternal environmental exposures such as infection [67], maternal diet [68,69], and smoking [69,70] can modify neonatal T cell function, although the mechanisms are not clear. Studies on environmental signaling in early life through dietary intake, despite the various controversies, suggest that these can modify the immune response to protect against allergy [71]. Maternal supplementation with omega-3 (n-/ω-3) long-chain polyunsaturated fatty acids (LC-PUFA; fish oil) during pregnancy modifies neonatal immune responses [68], in particular T cell function [68,69]. Recent evidence suggests that the *n*-3 LC-PUFA could mediate changes in developing patterns of neonatal T cell responses by influencing the levels of PKC isozyme expression. Of the PKC isozymes examined, only PKCζ was significantly higher in the fish oil group [60]. As PKCζ was shown to be an independent predictor of reduced subsequent allergic disease, this may present an important pathway through which antenatal *n*-3 LC-PUFA could have clinical effects in reducing allergic disease [72].

The results suggest that PKCζ expression is amenable to regulation by prenatal nutritional exposures, perhaps through epigenetic modifications such as DNA methylation or histone acetylation [73,74,75]. While in utero exposure to fish oil did not significantly affect T cell DNA methylation profiles, using epigenome-wide analysis of neonatal CD4^+^ T cells [76], it was found that the increase in PKCζ levels in CBTC caused by such supplementation correlated with modifications of histone acetylation at the PKCζ gene (*PRKCZ*) promoter level. The data suggest that PKCζ expression regulates the maturation of neonatal T cells, i.e., from a Th2 to Th1 phenotype [64]. CB CD4^+^ T cells obtained from the offspring of fish oil-treated pregnant women showed higher acetylation levels of H3 histones at the promoter of *PRKCZ*, the PKCζ encoding gene, corresponding to more transcriptionally permissive chromatin status and thus higher PKCζ expression [26,77,78,79,80,81]. This suggests that the effect of prenatal fish oil supplementation is at least partly mediated via epigenetic control of PKCζ synthesis.

A biochemical and epigenetic basis for development of neonatal immature T cells towards a propensity to give rise to either Th1 or Th2 type responses, and the consequent risk of allergy development in childhood and adulthood has emerged from these studies. It is evident that CBTC PKCζ levels play an important role in dictating this development. The PKCζ levels and most likely the Th cell type outcome can be altered by exposure to n-3 LC-PUFA, which appear to be working through an epigenetic mechanism of acetylation of histone H3 in the *PRKCZ* promoter. Thus, the PKCζ levels in CBTC are not only a potential biomarker for those that are at risk of developing allergy but may be an intervention target to prevent allergy development in the community. In relation to other biochemical pathways that may regulate the skewing of the Th cells is indoleamine 2,3-dioxygenase (IDO), which degrades tryptophan to kynurenine. Results have shown that there is a reduction in blood kynurenine metabolites in children from mothers who had received fish oil during pregnancy and this was correlated with lower risk of allergic asthma in the children [82]. Other studies have found that kynurenine metabolites can cause apoptosis of Th1 cells, without affecting Th2 cells [83]. This may explain a shift to Th1 in babies whose mothers were given fish oil when pregnant, although it has been reported that allergic children had reduced levels of IDO [84].

In conclusion, in utero and in early life, there is a high level of plasticity in T cell development, enabling some adaptation to the environment through modulation of the immune development, e.g., skewed towards a Th1 or Th2 cytokine phenotype, either directly or indirectly by, e.g., promoting development of ILC2 cells which in turn foster the Th2 skewing that may manifest as allergic disease development. PKCζ has been found to play a key role in the prevention of the Th2 skewing and its levels in naïve CBTC correlate negatively with the risk of developing allergy. Here we argued that a mechanism by which environmental signaling may modulate this immune response development is through the regulation of levels of T cell PKCζ. We used an example of a nutritional supplement that highlights our point, *n*-3 LC-PUFA, where the data suggest that the fatty acids protect against allergic sensitization and increase the level of PKCζ in CBTC. The findings also suggest that the *n*-3 LC-PUFA modify the CD4^+^ T cell PKCζ levels via an epigenetic mechanism.

## 3. Effects of Fatty Acids (FA) and Vitamins on Epigenetic Signatures and Their Relation to Allergies

### 3.1. FA

Epidemiological studies, animal models, and in vitro data have shown differential epigenetic landscapes in individuals exposed to suboptimal or undesired prenatal dietary exposures (i.e., nutritional deprivation in utero, maternal obesity, or maternal inflammatory diet) [85,86,87,88,89,90,91]. Of these, lipids have been of special interest among studies involving early epigenetic programming. High-fat diets during early life have been demonstrated to induce epigenetic changes at genome-wide level and in gene-specific loci (e.g., in genes associated with obesity, insulin resistance, and type 2 diabetes) [92,93,94,95,96,97,98], and to regulate the expression of certain miRNAs related with obesity and lipid metabolism [99,100]. Indeed, adequate nutrition with FA during the first thousand days of a child’s life is pivotal for healthy development and a healthy life [101].

Lipids exist as FA, acylglycerols, complex lipids (i.e., phospholipids and sphingolipids), isoprenoids (i.e., fat-soluble vitamins and steroids), and eicosanoids (i.e., prostaglandins and leukotrienes). More specifically, FA are amphipathic lipid molecules composed of a carboxyl group and an aliphatic (linear) hydrocarbon chain. Based on the length of the hydrocarbon chain, FA are classified as short-chain (<6 carbons; SCFA), medium-chain (6–12 carbons), long-chain (13–21 carbons), and very long-chain FA (when they have more than 21 carbons). Moreover, FA without double bonds are called saturated FA, as opposed to unsaturated FA that can have one (monounsaturated, MUFA) or more (polyunsaturated, PUFA) double bonds. The disposition of the double bonds determines if an unsaturated FA is categorized as cis or trans. Depending on the location of the first double bond, PUFA can be further classified as ω-3 and omega-6 (ω-6). Linoleic acid (a ω-6 PUFA) is the most abundant PUFA in our diet, mainly derived from plants, along with linolenic acid (a ω-3 PUFA). On the other hand, eicosapentaenoic acid (EPA) and docosahexaenoic acid (DHA), both ω-3 PUFA, are abundant in fish and fish oil [102,103]. Humans are incapable of synthesizing some FA like ω-linolenic acid and linoleic acid, and are thereby called essential FA. In the last decades, the dietary intake of FA has changed from those enriched in MUFA and PUFA to a diet with a high content in saturated FA and trans-FA. Currently, a number of studies support the view that FA promote epigenetic alterations that affect several metabolic and inflammatory pathways, contributing to the increase of non-communicable chronic diseases (reviewed by [104]). SCFA and other molecules influencing the evolution of allergic and other chronic inflammatory diseases can be obtained directly from the diet, or as metabolic products resulting of the fermentation by the intestinal microbiota of dietary fiber and other components [105,106,107,108,109,110,111]. In this chapter, we discuss the epigenetic modifications induced by FA obtained from dietary sources including those originated from the fermentation of non-digestible carbohydrates by the intestinal microbiome.

PUFA can induce epigenetic alterations both in vitro and in vivo [112,113,114]. For instance, perinatal maternal consumption of ω-linolenic acid correlates with the DNA methylation status of a gene encoding the mouse FA desaturase 2 (*Fads2*) in the livers of mothers and their progeny [115]. Moreover, some studies have underpinned the role of ω-3 PUFA availability during gestation and lactation in the epigenetic regulation of genes involved in nervous system development and its functions [116,117]. Similarly, PUFA have been shown to induce epigenetic changes with considerable impact in the immune system. Pre- and postnatal supplementation of pigs with ω-3 FA altered DNA methylation profiles in white blood cells of the offspring, affecting genes implicated in key biological processes, including inflammation, apoptosis, and oxidative stress [118]. Likewise, DNA methylation landscapes in human CB leukocytes varied between mothers with low, medium, and high ω-3 PUFA on erythrocyte membranes (a proxy of their ω-3 PUFA intake) [113]. Children of mothers who received DHA supplementation during pregnancy (versus placebo group) had differentially methylated regions, which occur in a gender-specific fashion, and are located in loci encoding for proteins related to immune function such as retinoic acid (RA) early transcript 1L (*RAET1L*) and lymphotoxin beta (*LTB*) [119]. Fish oil supplementation during infancy also induced DNA methylation changes at certain CpG sites in mononuclear cells, compared to sunflower oil supplementation [120]. The effects of ω-3 PUFA supplementation on the DNA methylation profiles of white blood cells has been also detected in adults [121].

PUFA modulate the immune system by regulating cytokine production [68,122,123,124,125] and have been implicated in the risk of allergic diseases. Fish oil supplementation in infants at high risk of atopy increased ω-3 PUFA levels and decreased allergen-specific Th2 responses, including IL-13 and IL-5 production, thus suggesting a potential protection against allergy [126]. In fact, several lines of evidence attribute a protective effect against the development of allergies and asthma to ω-3 PUFA consumption, especially during the perinatal stage [72,127,128,129,130,131,132,133,134,135]. The dosage, timing, and duration of ω-3 PUFA seem to be critical for this effect to take place [136,137]. Conversely, ω-6 PUFA (and a decreased ω-3-to-ω-6 ratio) has been generally considered as proinflammatory and associated with an increased risk of asthma and allergic conditions [127,130,134,136]. Although the mechanisms through which PUFA exert an influence on allergy are not fully understood, recent studies suggest that epigenetic changes might play a role. Fish oil and its bioactive component, ω-3 PUFA, are epigenetic modifiers of histone marks. For instance, fish oil supplementation affects histone acetylation levels in neonatal T cells in immune genes such as *PRKCZ*, IL-13 gene (*IL13*), and T-box 21 gene (*TBX21*) [64]. In another study, maternal fish consumption during pregnancy was associated with increased H4 acetylation in the CD14 molecule (*CD14*) gene in placentas, an effect that was mainly significant in those of female offspring [138]. Although fish intake involves the exposure to several compounds, based on current evidence it seems that those effects could be attributed to fish oil.

Another source of FA with demonstrated ability to induce epigenetic changes is extra virgin olive oil. Previous studies suggest that maternal olive oil intake as part of a Mediterranean diet is protective from wheezing in their offspring [139,140]. These effects are attributed to olive oil polyphenols, as well as long-chain ω-3 PUFA. More recently, it has been found that regular use of olive oil as a main cooking fat during pregnancy has been associated with increased H3 acetylation in the promoters of forkhead box P3 (*FOXP3*), IL-10 receptor subunit alpha (*IL10RA*), and IL-7 receptor (*IL7R*) genes, suggesting that prenatal intake of olive oil can affect placental histone acetylation in immune regulatory genes, and this supports previous studies suggesting the pro-acetylation effects of olive oil polyphenols [141,142]. In summary, consumption of olive oil and fish oil are regarded as factors with the ability to induce immune regulation which can in turn influence immune priming and allergen sensitization in the offspring [64,138,143]. Among all the studied loci, the *FOXP3* gene, encoding a master regulator of immune homeostasis, seems to be a prominent target of the epigenetic modifications induced by dietary FA and also by all-trans retinoid acid [144], microbial-derived butyrate [145], and propionate [146], and during the prenatal period by the intake of high fiber and acetate [147].

**Figure 5:**
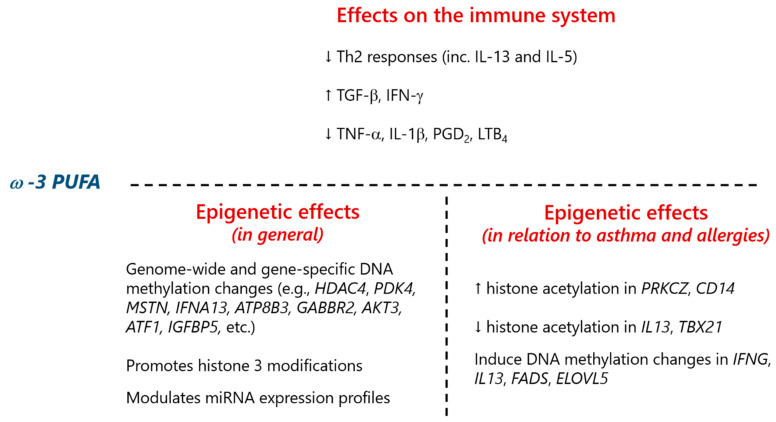
Summary of the effects of omega-3 polyunsaturated fatty acids (ω-PUFA) on epigenetic modifications related to immune function and allergy. For details, please refer to the main text, Chapter 3.1. *FA*. Th2 (cells), type 2 T helper (cells); IL, interleukin; TGF-β, transforming growth factor β; IFN-γ, interferon γ; TNF-α, tumor necrosis factor α; PGD2, prostaglandin D2; LTB_4_, leukotriene B_4_; *HDAC4*, histone deacetylase 4 gene; *PDK4*, pyruvate dehydrogenase kinase 4 gene; *MSTN*, myostatin gene; *IFNA13*, interferon α 13 gene; *ATP8B3*, ATPase phospholipid transporting 8B3; *GABBR2*, γ-aminobutyric acid type B receptor subunit 2; *AKT3*, AKT serine/threonine kinase 3; *ATF1*, activating transcription factor 1; *IGFBP5*, insulin like growth factor binding protein 5; miRNA, microRNA; *PRKCZ*, protein kinase C ζ gene; *CD14*, CD14 molecule gene; *IL13*, IL-13 gene; *TBX21*, T-box 21 gene; *IFNG*, IFN-γ gene; *FADS* (*1*/*2*), fatty acid desaturase (1/2) gene; *ELOVL5*, ELOVL fatty acid elongase 5 gene.

Besides histone modifications, ω-3 PUFA can also modify DNA methylation as a mechanism implicated in its immunomodulatory properties. An interventional study conducted in human infants revealed that gestational supplementation with ω-3 PUFA altered long interspersed nuclear element 1 (*LINE1*) repetitive sequences’ methylation in children of women who smoked during pregnancy. The modulated DNA methylation levels in the genes encoding IFN-γ (*IFNG*) and IL-13 (*IL13*) suggested an impact on Th balance (Th1/Th2) at this age [148]. In addition, maternal oily fish intake was associated with differential DNA methylation levels in FA desaturase 1/2 (*FADS1*/*2*) and ELOVL FA elongase 5 (*ELOVL5*), the genes encoding enzymes involved in PUFA biogenesis, and a decrease in *ELOVL5* mRNA [149]. Interestingly, methylation levels at certain CpGs of these genes showed a correlation with eczema and/or wheeze [149]. A summary of the effects of ω-3 PUFA on epigenetic modifications related to immune function and allergy is presented in Figure 5.

Other lipids also induce epigenetic modifications. Marchlewicz et al. identified long-chain FA, very long-chain FA, and acylcarnitines in maternal circulation that correlated with DNA methylation levels in the infant CB [150]. In addition, oleic acid (MUFA) reduced DNA methylation levels in the genes *Pparg* and *Cebpa* and at a genome-wide level [151,152]. Elaidic acid, a trans isomer of oleic acid, is able to alter the global DNA methylation landscape in vitro by inducing a pro-inflammatory and adipogenic gene expression program, which is different to that induced by oleic acid. These effects are observed in the offspring of elaidic acid-supplemented rats even 3 months after birth [153]. Trans fatty acids are also capable of altering the concentrations of miRNAs including hsa-miR-31-5p and hsa-miR-150-5p [154]. Moreover, hybrid palm oil (which is richer in oleic acid compared to African palm oil) has been associated with the regulation of several miRNAs (e.g., miR-488-3p, let-7b-5p, miR-15b-5p, and miR-17-5p-1) in the hepatic tissue of the common marmoset *Callithrix jacchus* [155]. The epigenetic changes induced by diets rich in oleic acid were not limited to genomic DNA but also affected mitochondrial DNA [156]. Palmitic acid is a saturated fatty acid regarded as pro-inflammatory, which affects DNA methylation, modifies histone acetylations, and modulates the expression of miRNAs (e.g., miR-181c) and long non-coding RNAs (e.g., BDNF-AS) [157,158,159,160,161,162]. Like palmitic acid, stearic acid enhances DNA methylation in macrophages [163]. Studies in rats treated with either coconut oil, sunflower oil, or olive oil revealed significant changes in the DNA methylation levels of the tumor necrosis alpha (TNF-α) gene (TNF) and thereby in TNF-α secretion [164]. Moreover, the adipose tissue from rats fed with coconut oil (the one with the highest proportion of saturated fatty acids, including palmitic acid [165]), exhibited the highest levels of TNF-α (both mRNA and secreted), as well as the lowest DNA methylation levels in the *TNF* promoter [164]. In contrast, cells treated with oleic acid or linoleic acid showed lower *TNF* mRNA levels [164].

In recent years, untargeted lipidomics have revealed that the number of lipids and their combinations is very complex [166,167]. Indeed, there are hundreds of different lipids, some of bacterial origin, which are extremely important for a healthy immune system during the perinatal period [168]. These include the microbial-derived SCFA that induce epigenetic changes implicated in the susceptibility to allergic diseases. Acetic acid, butyric acid, and propionic acid are the most abundant SCFA and are regarded as “postbiotics” [169]. They are produced by the fermentation of nondigestible carbohydrates contained in dietary fiber and are largely influenced by the type of diet and gut microbiome composition. SCFA are found within the first hours after birth, as evidenced in stool samples of newborns [170]. SCFA are mainly produced in the large intestine and are used as an energy source by colonocytes. In addition, they play an important role in keeping pathogenic bacteria under control and maintaining intestinal integrity and health [171,172,173]. A proportion of these SCFA is absorbed by the portal vein, carried to the liver, metabolized, and then systemically distributed via the bloodstream. Some SCFA activate free FA receptors 2 (FFAR2/GRPR43) and 3 (FFAR3/GPR41) on intestinal epithelial cells, leading to mitogen-activated protein kinase signaling and rapid production of chemokines and cytokines involved in protective immunity. On the other hand, SCFA acting on receptors like GPR43 or hydroxycarboxylic acid receptor 2 (HCAR2/GPR109A) mediate oral tolerance. SCFA are also well recognized for inhibiting histone deacetylases (HDAC) and modifying gene expression by enhancing histone acetylation [174,175,176,177,178,179]. Butyric acid, in particular, inhibits class I and most of class II HDAC [180]. SCFA promote histone acetylation of *FOXP3* (encoding a key transcription factor for Tregs development), stimulating its expression [145,147]. SCFA also induce Treg proliferation not only in the colon but in other tissues like the skin [107,146,181,182]. This is very critical because Tregs are essential for the generation tolerance to allergens and the containment of immune response. Using a murine model, Nakajima et al. showed that offspring of high-fiber diet-fed mothers have high levels of SCFA and high frequencies of Tregs [183]. Inhibition of histone deacetylase 9 (HDAC9) is of special relevance for the SCFA-induced, *Foxp3*-dependent suppressive activity of Tregs [184,185]. Indeed, *Hdac9^-/-^* mice, exhibit resistance to the development of inflammatory conditions like colitis and allergic airway disease [147,186]. Beyond inducing Treg development, SCFA also can reduce type 2 airway inflammation, regulate mast cell generation and function, and reduce eosinophil trafficking and survival [185,187,188,189,190,191,192]. In line with this, data collected from animal models and human observational studies have shown that SCFA during pregnancy and early life has an impact on the occurrence of allergic diseases [193,194,195,196]. Roduit et al. measured fecal SCFA levels in stool samples of one-year-old children and found that butyric acid and propionic acid were inversely associated with atopic sensitization, as well as with allergic outcomes later in life [197]. In addition, the infants whom developed allergic diseases had gut bacterial species with fewer genes encoding enzymes needed for fermentation of butyric acid [198]. SCFA could be useful in the prevention of allergic diseases via high-fiber diets or direct oral administration, and once measured could serve as biomarkers to predict allergic diseases early in life [179,197,198,199].

**Figure 6:**
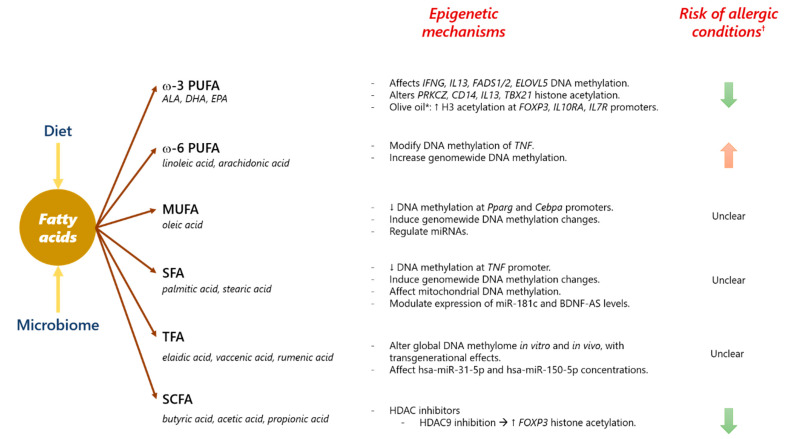
Epigenetic effects of dietary- and microbiome-derived fatty acids and their relationship with allergic diseases. For details, please refer to the main text, Chapter 3.1. *FA*. ^†^ Regarding the perinatal period. As mentioned in the main text, the impact of a given lipid on the risk of allergic conditions is not univocal. More research is needed to further clarify the links between these lipids in early life and the risk of allergic conditions. *Olive oil is known as an important source of *n*-3 polyunsaturated fatty acids (PUFA). ALA, alpha linolenic acid; BDNF-AS, brain-derived neurotrophic factor antisense RNA; CD14, CD14 molecule; *Cebpa*, CCAAT/enhancer binding protein alpha gene; DHA, docosahexaenoic acid; ELOVL5, ELOVL fatty acid elongase 5; EPA, eicosapentaenoic acid; *FADS1/2*, fatty acid desaturase 1/2 gene; *FOXP3*, forkhead box P3 gene; HDAC, histone deacetylases; HDAC9, histone deacetylase 9; *IFNG*, interferon gamma gene; *IL7R*, interleukin 7 receptor; *IL10RA*, interleukin 10 receptor subunit alpha; *IL13*, interleukin 13; miRNAs, micro RNAs; MUFA, monounsaturated fatty acids; *Pparg*, peroxisome proliferator activated receptor gamma gene; *PRKCZ*, protein kinase C zeta gene; SCFA, short-chain fatty acids; SFA, saturated fatty acids; *TBX21*, T-box transcription factor 21 gene; TFA, trans fatty acids; *TNF*, tumor necrosis factor.

In conclusion, lipids either from dietary sources or produced by the gut microbiome play a key role in the developing immune system. Indeed, exposure to certain lipids during the perinatal period can shift the balance towards allergic sensitization and increase the risk to allergic diseases (Figure 6). This association between fatty acids and allergy is largely explained by multiple epigenetic mechanisms affecting inflammatory vs. immunomodulatory pathways. Albeit most research has been focused on PUFA and SCFA, there are many other lipids including MUFA and saturated fatty acids that might also affect the immune system and require further research. Untargeted analyses of lipids are urgently needed to elucidate their effects as risks factors for allergy and to pave the way for their clinical application in personalized medicine. The use or avoidance of certain fatty acids can provide benefits in the prediction and/or prevention of allergic conditions. Further research on the exact epigenetic mechanisms behind the relationship of fatty acids and allergic diseases is a pressing need.

### 3.2. Vitamins

Vitamins are well known to be critical for immune system functioning. For instance, vitamins D and A are widely known for their immunomodulatory properties [200,201,202]. Moreover, the susceptibility to allergic entities can be influenced by perinatal consumption of vitamins [69,203,204,205,206,207,208,209,210,211,212,213,214,215]. Several studies have uncovered the role of vitamins in epigenetic programming, particularly during early life. The best demonstrated effects are those of maternal dietary methyl donor intake (and cofactors) on DNA methylation in both fetus and placenta [216]. Indeed, plasma folate levels in mothers correlate with offspring DNA methylation, especially in genes related to neural tube defects and other birth defects, neurological functions, growth, and embryonic development [217]. Maternal vitamin D supplementation during pregnancy and breastfeeding modifies the DNA methylation landscape in their breastfed infants, mainly in genes involved in collagen metabolism and regulation of apoptosis [218]. In addition, maternal plasma levels of vitamin D along with ancestry affect DNA methylation in infants [219]. Murine models showed that vitamin D deficiency during pre- and postnatal development induces changes in the methylome that can persist throughout many generations [220,221]. More specifically, maternal vitamin D status, as well as gestational supplementation with this micronutrient, inversely correlate with perinatal DNA methylation at the retinoid X receptor alpha (*RXRA*) gene [222,223].

Furthermore, cell culture experiments have underscored the epigenetic effects of vitamin A through demonstrating its influence on chromatin structure [224]. For instance, all-trans retinoid acid, a form of vitamin A, induces alkaline phosphatase expression via chromatin remodeling of its promoter [225].

Regarding the B-complex vitamins, an analysis of the relation between genome-wide DNA methylation in blood leukocytes and dietary folate and vitamin B_12_ showed differential methylation in association with the intake of these micronutrients [226]. Folate inversely associated with DNA methylation of at least 73 genomic regions, while vitamin B_12_ intake was associated with differential methylation in at least 29 genomic regions [226]. Maternal vitamin B_6_ concentrations have been positively associated with DNA methylation at the long non-coding RNA maternally expressed gene 3 (*MEG3*) in the progeny [227]. Concerning vitamin C, it has been shown to reverse DNA methylation changes associated with maternal smoking during pregnancy [228].

**Figure 7:**
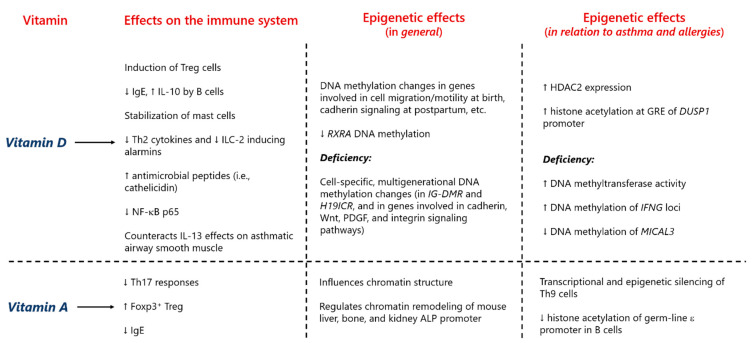
Summary of the epigenetic effects of vitamins D and A potentially relevant for allergy predisposition. For details, please refer to the main text, Chapter 3.2. *Vitamins*. Treg (cells), regulatory T (cells); IgE, immunoglobulin E; IL, interleukin; Th2 (cells), type 2 T helper (cells); ILC-2, type 2 innate lymphoid cells; NF-κB p65, a p65 subunit of the nuclear factor κB transcription complex; Th17 (cells), type 17 T helper (cells); Foxp3, forkhead box P3; *RXRA*, retinoid X receptor alpha gene; *IG-DMR*, intergenic differentially methylated region; *H19ICR*, H19 imprinting control region; PDGF, platelet-derived growth factor; ALP, alkaline phosphatase; HDAC2, histone deacetylase 2; GRE, glucocorticoid response element; *DUSP1*, dual specificity phosphatase 1 gene; IFN-γ, interferon γ; *MICAL3*, microtubule-associated monooxygenase, calponin and LIM domain-containing 3; Th9 (cells), type 9 T helper (cells).

In recent years, it has been found that epigenetic mechanisms play a significant role in the relation between vitamins and allergy. For instance, the RA signaling pathway has been shown to suppress the transcriptional and epigenetic program (with a particular effect on the IL-9 gene) of Th9 cells, an important subset in the pathogenesis of atopy [229]. Additionally, RA represses IgE class-switching recombination via inhibition of histone acetylation of the germ-line ԑ promoter [230]. Furthermore, evidence substantiates that gestational vitamin D deficiency decreases the Th1/Th2 ratio and IFN-γ production, while increasing IL-4 concentration [231]. Moreover, as a result of maternal vitamin D deficiency, the activity of DNA methyltransferase is heightened, as well as the methylation of the IFN-γ locus, effects that can be reversed by vitamin D supplementation during pregnancy [231]. Prenatal vitamin D deficiency positively associates with the risk of AD, an effect that is mediated at least in part by a diminished DNA methylation of microtubule-associated monooxygenase, calponin, and LIM domain-containing 3 (*MICAL3*), a gene related to the generation of reactive oxygen species [232]. However, another study evaluating CB vitamin D levels in relation to allergic diseases and DNA methylation delivered different results [233]. On the other hand, a comprehensive epigenetic and gene expression analysis performed in a cell line of bronchial epithelial cells from asthmatic patients showed that vitamin D in fact modifies the epigenetic landscape of these cells [234,235]. This combined epigenetic and transcriptomic approach allowed for the proposal of candidate genes (e.g., *DUSP10* encoding dual specificity phosphatase 10 and *SLC44A1* encoding solute carrier family 44 member 1) for asthma and viral infection susceptibility [234,235]. In monocytes from asthma patients, vitamin D pretreatment increased glucocorticoid receptor binding to the *DUSP1* (a gene encoding dual specificity phosphatase 1, a protein responsible for the inhibition of pro-inflammatory cytokines production) promoter and enhanced histone H4 acetylation at the glucocorticoid response elements of this genomic region [236]. Moreover, vitamin D treatment increased HDAC 2 (HDAC2) expression and in turn decreased airway inflammation and NF-κB p65 (a p65 subunit of the nuclear factor κB transcription complex) expression in a murine model of ovalbumin-induced allergic airway inflammation, mimicking human asthma [237]. All these data provide plausible biological explanations for the protective effect of vitamins, mostly during the perinatal stage, for atopic entities and asthma. A summary of the epigenetic effects of vitamins A and D that could be relevant for allergy predisposition is presented in Figure 7. 

## 4. Effects of Microbes and Parasites on Epigenetic Signatures and Their Relation to Allergies

**Figure 8:**
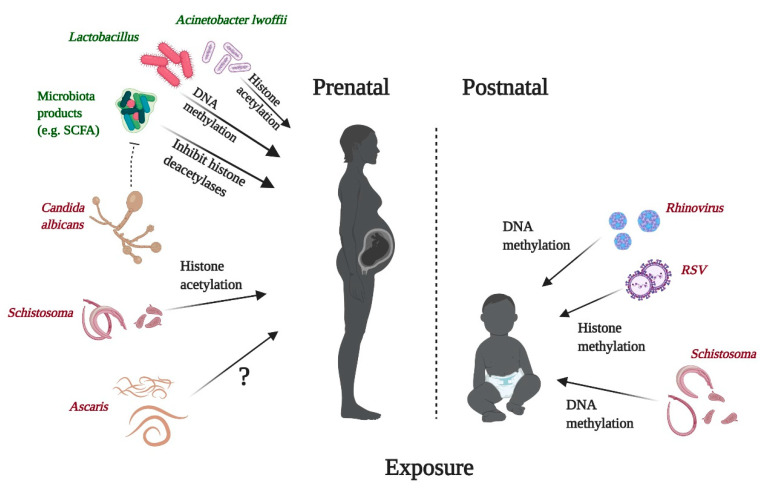
Perinatal epigenetic effects of microbes and parasites in the context of allergies. For details, please refer to the main text, Chapter 4. *Effects of microbes and parasites on epigenetic signatures and their relation to allergies*. SCFA, short-chain fatty acids; RSV, respiratory syncytial virus. Created with BioRender.com.

Early life microbial exposure to bacteria, viruses, parasites, or fungal components can impact immune responses, thus contributing to the risk of allergic disease development throughout life. Those influences can be of a pro- or anti-allergic character depending on several factors, especially the type of the microbe and the timing and location of the exposure [2], and are mediated by complex gene–environment interactions modulating epigenetic modifications in the genes involved in the pathogenesis of allergy [238,239,240]. The epigenetic effects of microbes on the immune system can be mediated metabolically through SCFA, as in the case of some gut bacteria, but many other modes of action are also known. In addition, considering the pivotal role of gut bacteria and the metabolites synthesized by them from dietary components such as SCFA, nutrition can strongly modify the effects of microbes on host immunity and health. Furthermore, microbes and their elements or metabolic products are integral elements of some foods, especially of more traditional origin [2,241]. Perinatal and some other epigenetically mediated effects of microbes on human immunity and health are shortly outlined below and summarized in Figure 8 and Table 1.

### 4.1. Bacteria

For many years, the uterus has been considered as a sterile womb, with the fetus being fully protected from microbial colonization. However, some recent studies have questioned this hypothesis, demonstrating that bacterial colonization occurs not only after the birth, but may begin already in utero [252,253]. The diversity and composition of the microbiome developing from infancy to childhood depends on multiple influences, with the interaction between microbes and the host immune system occurring during and beyond this period being functionally involved in the development of chronic inflammatory and/or immunometabolic disorders later in life [254,255,256]. This is also true for allergies, as demonstrated by studies in mice perinatally treated with antibiotics showing that the permanent changes in gut microbiota early in life can result in higher susceptibility to the subsequent development of atopy or allergic asthma [257,258].

Despite many studies demonstrating the dynamics of the human microbiota early in life, still relatively little is known about the influence of microbiota on epigenetic signatures and its immunopathological consequences. Compared to the placebo group, maternal supplementation with *Lactobacillus reuteri* during pregnancy resulted in altered DNA methylation patterns in CD4^+^ T cells, especially at birth, with DNA of the babies who received probiotics characterized mostly by lower methylation, indicating transcriptional accessibility [242]. A farm bacterium *Acinetobacter lwoffii* has been described as a differential environmental factor between two communities characterized by lifestyles varying with regard to features affecting the microbial exposure such as farming or westernization [259]. Modeling the continuous exposure to the traditional farming, microbe-rich environment through repeated intranasal administration of *A. lwoffii* to pregnant mice revealed the presence of the transgenerational effect, with the progeny being protected against the development of eosinophilic asthma [260]. Subsequently, this transmaternal protective effect of *A. lwoffii* was demonstrated to be at least partly mediated through stabilization of histone H4 acetylation at the *IFNG* promoter of CD4^+^ T cells deriving from spleens of the offspring [240].

Although the mechanisms by which specific microbes may affect the epigenome are not yet fully understood, it is known that some of them refer to their metabolic activity [261]. SCFA, represented by acetate, propionate, butyrate, and others, are the major product of intestinal bacterial fermentation of ingestible foods, e.g., poly- or oligosaccharides [262,263,264,265,266]. As the synthesis of SCFA is highly dependent on the gut microbiota, it can be strongly affected by an antibiotic therapy [267]. As it provides appropriate substrates, the diet is also an important factor affecting SCFA production in the gut [268].

Independently of the effects exerted by SCFA through their receptors, GRPR43 and GPR41 [269,270], inhibition of histone deacetylation is a very important mechanism by which SCFA influence human immunity and thus immunopathology [262,263,264,265]. For example, mice fed with a high-fiber diet developed a distinctive gut microbiota producing higher amounts of acetate. This acetate was able to increase the percentage and activity of Treg cells by enhancing the histone acetylation status of the *Foxp3* promoter through HDAC9 inhibition [147]. In addition, a high-fiber or -acetate diet ameliorated allergic airway inflammation in a murine model mimicking human asthma, as well as in the offspring, thus demonstrating the existence of the transmaternal transmission of the epigenetic effect [147]. In addition, butyrate inhibits histone deacetylases and, in this way, influencing at least partly the activation of the transcription factor NF-κB [244]. Stimulation of human and mouse mast cells with butyrate and propionate revealed a reduction in their activation and degranulation. These effects were related to HDAC inhibition, but not signaling through GPR41, GPR43, and peroxisome proliferator-activated receptor (PPAR) pathways. Furthermore, it was observed that pretreatment with butyrate inhibits human mast cell activation by downregulating the genes essential for this process such as the tyrosine kinases BTK (Bruton tyrosine kinase), SYK (spleen-associated tyrosine kinase), and LAT (linker for activation of T cells). While butyrate exerted an increase in global histone H3 lysine 27 (H3K27) acetylation, it decreased locus-specific H3K27 acetylation at the promoter regions of the genes encoding BTK, SYK, and LAT [243]. Although SCFA appear to be the most recognized histone acetylation modulators of bacterial origin, products of phytate microbial metabolism such as inositol-1,4,5-trisphosphate were found to stimulate the activity of HDAC3 to promote epithelial repair in the gut [271]. Histone acetylation induced by butyrate produced by the *Clostridial* cluster, *Anaerostipes*, and *Eubacterium*, but also DNA methylation and N6-methyladenosine (m6A) modification of mRNA increased by folate synthesized by species such as *Lactobacillus* and *Bifidobacterium*, enhance the development and immune balance of the intestine [272].

Polyphenols are dietary products deriving from plant food that are characterized by the presence of phenol groups in their structure and their antioxidant properties [273,274]. In plants, polyphenols have been shown to be a part of defense mechanisms against pathogens and, currently, they have been gaining more and more visibility due to their modulating effects on human health [273,275]. The role of these compounds in the context of gut flora can be seen from at least two major points of view such as the modulatory effects exerted by them on the intestinal microbiota and the impact of the intestinal microbiota on bioavailability of polyphenols. Indeed, the polyphenols seem to alter the gut microbiota, with changes in the proportion of multiple bacteria such as lactobacilli, bifidobacteria, *Bacteroidaceae*, clostridia, *Staphylococcus* genera, and others [276,277,278]. On the other hand, intestinal flora can increase the otherwise rather low bioavailability of dietary polyphenols. The resulting compounds can subsequently exert epigenetic effects on the host, for instance, DNA methyltransferase (DNMT) inhibition by resveratrol, HDAC inhibition by sulforaphane, histone acetyltransferase (HAT) inhibition by (-)-epigallocatechin-3-gallate (EGCG) in green tea, as well as regulation of miRNAs by genistein [47]. Beneficial effects of the perinatal supplementation of polyphenols mediated through the epigenetic changes triggered by them in the host have been observed, such as contribution to chronic disease prevention [279] and the modulation of the central nervous system development [277,280]. Similar roles of polyphenols have been suggested for allergies as well [281].

Regarding the upper respiratory tract microbiota, its composition in early life has been shown to contribute to the development of AR in children, partly through changes in DNA methylation patterns in the upper airway mucosa [282].

In conclusion, it can be seen how, through epigenetic mechanisms, microbiota or their metabolic products are capable of leaving immune signatures in the host and the progeny, including those allergy-related. Although most of the relevant observations have been made for adaptive immune responses, some progress has also been achieved in the case of innate immunity, prompting further research in this area.

### 4.2. Viruses

As observed in human studies and mouse models, respiratory viral infections are not only capable of enhancing the immune response to allergens but also of increasing exacerbation rates and even the incidence of allergic diseases [283,284]. The risk of subsequent wheezing, atopy, and asthma in infants is increased following respiratory infections caused by viral agents such as respiratory syncytial virus (RSV) or human rhinoviruses (RVs), which are most prevalent in early life [285,286,287]. Virus-induced respiratory wheezing symptoms have been shown to correlate with the genetic variation at the 17q21 locus [288,289]. In a COPSAC_2010_ birth cohort study, an association between the chromosome 17q21 variants and RV- but not RSV-related wheezing illnesses was observed. Moreover, the expression of *ORMDL3* (encoding ORMDL sphingolipid biosynthesis regulator 3) and of *GSDMB* (encoding gasdermin B)*,* the 17q21 genes related to asthma, was significantly higher in RV-stimulated compared to the unstimulated peripheral blood mononuclear cells (PBMCs) [289]. A part of the 5′-untranslated region (5′-UTR) of *ORMDL3* is in CD8^+^ T cells less methylated than in other leukocyte subtypes, suggesting that DNA methylation differences in *ORMDL3* might promote T-lymphocyte-driven inflammation. Increased mRNA expression, together with DNA methylation changes in CD8^+^ T cells, can make some individuals more susceptible to a dysregulated inflammatory response mediated by CD8^+^ T cells upon viral infections. These observations could explain the associations between *ORMDL3* alleles and asthma in the context of personal antecedents of RV-related wheezing illness as well as with early-onset asthma phenotypes [290].

Furthermore, the development of atopic asthma following early life RV-induced wheezing was associated with DNA methylation changes at several asthma susceptibility loci, such as the promoter region of the SMAD family member 3 gene (*SMAD3*) in chromosome 15q22.33 and introns of the D-aspartate oxidase (*DDO*)/ methyltransferase like 24 (*METTL24*) genes in the 6q21 locus [246]. Another study revealed that IFN-γ-enriched milieu enhances the ability of respiratory epithelium to protect from RSV infection, possibly via epigenetic modification of *RIG-I* (*DDX58*), a gene encoding DExD/H-box helicase 58, and a resulting increase in its expression [291]. RSV infection was found to upregulate the expression of lysine demethylase 5B (KDM5B or JARID1B), a H3K4 demethylase, in dendritic cells (DCs). Experimental reduction KDM5B activity led in turn to enhanced Th1 and reduced Th2 responses [245]. Moreover, in airway epithelial cells, the non-structural protein 1 (NS1) of RSV was able to interact with the histone H2BD leading to its ubiquitination. A resulting induction of homeobox (*HOX*) gene expression might possibly contribute to the abnormal lung development and asthma predisposition [292].

Altogether, these findings demonstrate that viral respiratory infections can generate permanent epigenetic marks in genes associated with asthma, which in the long term can predispose to the disease development.

### 4.3. Parasites

In developing countries and the tropics, parasitosis still constitutes a public health problem and is a part of the ecosystem that can generate epigenetic modifications in its host. Parasite sensitization has been shown to start early in life. It was found that, in mothers with ascariasis, there is an in utero sensitization of newborns to *Ascaris lumbricoides*, with a greater frequency of CD4^+^ T cells producing IFN-γ and IL-4 in CB from newborns of infected mothers, showing that the immunological effects of infection develop in the fetus [293]. In a study of a cohort from the tropics, it could be observed how sensitization to *Ascaris* in mothers can influence total IgE levels in the child, assuming a possible in utero modulation of the Th2 response [294].

The influence of parasites on offspring phenotype is a topic that has been discussed for more than 10 years [295]; however, their epigenetic effects have been poorly investigated. In a longitudinal study, children with active *Schistosoma haematobium* and *A. lumbricoides* infection had in their CD4^+^ T cell DNA methylation signatures concordant with an inhibition of the Th1 immune phenotype. For schistosomiasis, but not ascariasis, six months after anti-helminthic treatment, the CD4^+^ T cell immune phenotype demonstrated an increase percentage of IL-4 producing and decreased percentage of TNF and IFN-γ producing antigen-specific CD4^+^ T cells compared to uninfected controls [248]. Consistently, in mice being born or breastfed by a mother infected with *Schistosoma mansoni*, the expression of HDACs was induced. In addition, gestation or breastfeeding seem to favor various IL-10-dependent pathways, but not cells with a Treg phenotype [296]. Furthermore, in another mouse study, chronic schistosomiasis during the pregnancy led to the Th2 differentiation impairment in the offspring through reduced levels of histone acetylation at the promoter of the gene encoding IL-4, as observed in naïve T cells [249]. Studies on in utero epigenetic effects of maternal *A. lumbricoides* infection and their immunological consequences are, however, still missing.

Immune response to helminths and allergic sensitization and inflammation share type 2 immunity mechanisms. It has been demonstrated in mice that maternal immune response to helminth infection during pregnancy determined offspring susceptibility to allergic airway inflammation [297]. Furthermore, Zakzuk et al. reported epigenetic changes in PBMCs from subjects infected with *A. lumbricoides*. These changes occurred in key genes of the type 2 immune response. Histone acetylation levels of *IL13* were inversely correlated with egg worm parasite burden and of the IL-4 (*IL4*) and chitinase 3-like 1 (*CHI3L1*) genes were associated with IgE levels to *A. lumbricoides*. In addition, the IgE response to house dust mite (HDM) was associated with histone acetylation of the TNF superfamily member 13b gene (*TNFSF13B*) encoding B cell activating factor (BAFF) [247]. It has been suggested that products derived from parasites may have an immunoregulatory and therapeutic effect in inflammatory diseases like allergies [298]. These products can generate epigenetic modifications in their targets. Innate immune cells such as macrophages trained in vitro with an extract of *Fasciola hepatica* had enhanced anti-inflammatory properties, which could be reverted by inhibitors of DNA methylation. In addition, macrophages from mice treated with an extract of *F. hepatica* or pretreatment with such an extract ameliorated the experimental autoimmune encephalomyelitis in a murine model. Together, those observations suggested that epigenetic modification by helminth products that renders them immunosuppressive may partly explain the reduced susceptibility to chronic inflammatory disorders [250].

### 4.4. Fungi

Colonization of the infants by fungi starts after the birth, and involves a number of genera, predominantly of *Cladosporium, Cryptococcus, Saccharomyces, Candida,* and *Malassezia* [239,299,300]. While most of the relevant studies focused on the bacteria-related epigenetic changes in asthma, the effects of the fungi, an important subgroup of human microbiota, referred to as mycobiota, have been left relatively unattended. Still, several recent studies have revealed the relationships between fungi and the health outcomes such as allergies, infections, and metabolic disorders, both in adults and in children [299]. Early-life gut fungal colonization with *Candida* and *Rhodotorula* was linked to atopy and asthma [301]. The fungal composition of the skin differs depending on the host age and sex, with children having more fungi characterized by a greater diversity and domination of *Malassezia* [239]. This genus has been shown to be an epigenetic regulator of the genes encoding IL-8 and β-defensin in human keratinocytes [251]. Furthermore, in mice, the *Aspergillus fumigatus* exposure during late pregnancy demonstrated an association with the low levels of IgE and eosinophils in the lung of the progeny. These protective effects of *A. fumigatus* correlated with the changes in CpG methylation in the promoters of the genes encoding IFN-γ and IL-4 [302].

Overall, the findings on the epigenetic changes related to the colonization with certain fungi only allow us to make some assumptions of potential mechanisms and generate the resulting hypotheses. In addition, due to the limited character of the available data, generalization or extrapolation of the findings to other organs or disorders may be difficult and thus further studies are needed.

## 5. Effects of Breastfeeding on Epigenetic Signatures and Their Relation to Allergies

### 5.1. Breastfeeding in Relation to AD, Food Allergy, and Allergic Asthma

Human milk is the normative standard for infant feeding and an optimal source of nutrition to achieve optimal growth, development and health of the neonate. Because of its dynamic nature, human milk changes in time to meet infants’ requirements as he/she grows. It comprises of a wide range of immunological nutrients and bioactive components, such as PUFA, SCFA, oligosaccharides, proteins, free amino acids, immunoglobulin A (IgA), micronutrients, anti-microbial peptides, vitamins, human milk microbiota, and many more, which are all important for building the immature gastrointestinal mucosa, the central nervous system, endocrine system, and the development and maturation of the infants’ innate and adaptive immune system [303,304,305,306]. Evidence shows a positive impact of breastfeeding on the infants’ anti-inflammatory response [307] which suggests that breastfeeding protects against infant morbidity, respiratory infections, and diarrhea during early life (infancy) [308,309,310,311,312].

The health benefits of human milk are widely accepted and it is recommended as the sole nutrition by the World Health Organization (WHO) for the first 6 months of life, continued with the introduction of complementary foods up to two years of age or beyond. These recommendations are in part based on initial studies of Saarinen et al. and Muraro et al., concluding that breastfeeding is prophylactic against certain atopic diseases—including atopic eczema, food allergy, and respiratory allergy—throughout childhood and adolescence [313,314,315]. Additionally, there is growing evidence that individuals who are breast-fed show lower risk for non-communicable diseases such as metabolic disease outcomes later in life (i.e., type 2 diabetes and obesity) [316]. Many studies have investigated the association between breastfeeding, bioactive human milk components, and allergic disease (Table 2). Recent studies suggest that human milk extracellular vesicles (EV) also contribute to the short- and long-term benefits of breastfeeding [317,318]. Breastfeeding appears to have a positive effect on gut and respiratory health; however, results are inconclusive, not in the least because of the definition of breastfeeding which includes exclusive breastfeeding, mixed feeding of breastmilk supplemented with infant formula, and their timeframes [319,320]. Studies investigating the long-term effects of breastfeeding on food allergy [321,322] and allergic asthma [323,324,325,326] showed no or even an increased risk in children who were breastfed. Systematic reviews and meta-analyses have concluded an overall protective effect of breastfeeding against allergic asthma [327,328]. Dogaru et al. found that breastfeeding was associated with a 22% reduced risk of asthma, with the strongest effect observed at 2 years of age. Lodge et al. showed a 10% reduced risk of asthma at 5 to 18 years. Although the limitations of these studies are recognized [329], the studies are supported by findings on the protective effects of breastfeeding on AD [314,327,330], manifestations of food allergy [313], and allergic asthma later in life [327,328,331,332,333]. The inconsistencies between studies are most likely due to differences in duration of breastfeeding, the amount of milk given, genetic predisposition, methodology, and differences in the composition of human milk between individuals and across populations [304,334,335]. Several studies have shown that milk composition is reflected by the maternal nutritional status. Supplementation regimes or variation in maternal diet lead to differences in human milk composition which contributed to the course of allergic disease [134,252,304,334,336,337,338,339,340]. In addition, ethnicity and environmental factors, i.e., smoking, having pets, and geographic location, can influence human milk composition. Interesting observations have been summarized regarding the composition of human milk of lactating woman from farming and urban environments. Exposure to a farm environment is associated with higher concentrations of transforming growth factor β1 (TGFβ1) and IL-10 in breast milk when compared to exposure to an urban environment [341]. Like human milk, raw cow’s milk is unprocessed and thermal processing of milk affects conformational changes to whey proteins, one of the major protein fractions in bovine milk. In an analogy to breast milk, numerous epidemiological studies have shown that the consumption of raw cow’s milk reduces the risk of allergic diseases as well [342]. These epidemiological findings were recently confirmed by causal evidence in pre-clinical animal models and a human pilot study showing that allergic children could tolerate raw milk better than pasteurized shop milk by showing less allergic symptoms upon drinking raw milk [343,344,345].

An ongoing debate concerns the role of dietary proteins in human milk and the risk for developing food allergy. Maternal allergen consumption during pregnancy and breastfeeding has been thought to control allergen sensitization in the offspring. Important studies by Ohsaki et al. and Verhasselt et al. showed that maternal ovalbumin sensitization protected the offspring against food-induced anaphylaxis and specific IgE. This protection was mediated by maternal IgG and ovalbumin immune complexes transferred via mother’s milk [346]. This and other scientific findings [336,338,347,348] supported the guidelines against maternal diet restriction during pregnancy and lactation [349].

In conclusion, it has been shown that human milk can be described as a combination of bioactive immune modulatory components with the putative capacity to modulate allergic disease. Beneficial effects of breastfeeding on human health can be highly influenced by environmental factors.

### 5.2. Breastfeeding, Epigenome, and Allergic Disease

The mechanisms responsible for the beneficial effects of breastfeeding on health in childhood and adulthood remain largely unknown. Maturation of the gut microbiome by bioactive milk components (i.e., milk oligosaccharides, IgA) is considered an important factor in the health benefits of breastfeeding. Nutritional exposures have been shown to regulate gene expression in adults by epigenetic processes and have led to the hypothesis that the beneficial effects of early life nutrition are induced at least in part by epigenetic mechanisms such as DNA methylation, histone modifications, or miRNAs [2,26,376,377]. Most studies on the effect of early-life nutrition on the epigenetic regulation of genes have focused on DNA methylation. Increasing evidence show that DNA methylation processes contribute to the positive effects of breastfeeding in relation to the development of non-communicable diseases, such as obesity [316,378,379,380,381]. In the next paragraph, we discuss the current knowledge on the role of epigenetics in the reduced risk for allergic disease. Early environmental exposures influence infants’ gene expression and developmental pathways during critical periods of pre- and postnatal development. These changes are thought to induce permanent changes in disease susceptibility and severity. Epigenetic regulations play a pivotal role in the pathophysiology of allergic diseases [2,27,138,143,382,383]. However, studies on breastfeeding and the role of epigenetic mechanism in the imprinting effects of breastfeeding or its components in relation to allergic diseases are very limited. Selected human milk components related to epigenetic mechanisms are discussed in the next paragraphs.

### 5.3. TGFβ

Several small studies have shown associations between breastfeeding and DNA methylations. A recent paper of Sherwood et al. showed in an epigenome wide study on the association between duration of breastfeeding with DNA methylation in children [384]. In particular, the hypermethylation of the gene encoding sorting nexin 25 (SNX25) is interesting in relation to allergic disease [384]. An increase in promoter methylation leads to a decrease in gene expression and consequently to downregulation at the protein level. SNX25 has been shown to downregulate TGFβ [385]. TGFβ is present in human breastmilk and is a regulatory cytokine connected to immune regulation and inflammation. TGFβ has been observed to be involved in the pathophysiology of allergic disease, although evidence is not a clear cut [335,357,386]. These inconsistent findings might be due to the rapid decline in TGFβ levels during lactations, with the highest levels in colostrum [335]. An important key regulatory role of TGFβ is the promotion of IgA, which positively influences the gut microbiome, another factor in the regulatory properties of human milk in the prospect of allergic disease [339,387]. In addition, the essential role of TGFß in Treg induction cannot be ignored [2,10].

### 5.4. MiRNAs

Human milk is one of the richest sources of miRNAs, which are mentioned as one of the important epigenetic mechanisms underlying the beneficial effects of human milk in the breast-fed infant. A single miRNA can bind and regulate multiple genes and affects the immune system by regulating T and B cell development, differentiation of dendritic cells, and the release of inflammatory cytokines, making them an important player in the health benefits of breastfeeding [388]. Specifically, miRNAs present in human milk during the first 6 months of breastfeeding, miR-155, miR-181a, miR-17, miR-150, and miR-223 [389], are associated with the development of the innate and adaptive immune system. A study by Simpson et al. measured a group of highly expressed miRNAs including miR-148a-3p, miR-22-3p, miR-30d-5p, let-7b-5p, and miR-200a-3p in 54 human milk samples collected 3 months postpartum [390]. However, authors observed no association between these specific miRNAs in the prevention of AD at two years of age in an intervention study with perinatal probiotics’ supplementation [390,391]. The presence of miRNAs in milk is not limited to humans. In addition, cow’s milk, a regular early nutrition of newborns, contains multiple miRNAs. Some of these protective miRNA’s are involved in obesity, type 2 diabetes, cancer, or Alzheimer’s disease [377]. However, the evidence for milk’s allergy-protective mode of action for miRNAs is still limited. The finding that miRNAs in cow’s milk promote regulatory T cells suggests an import role for miRNAs in allergic disease by their (indirect) effect on Th-2 mediated sensitization to food or inhalant allergens [377]. This is supported by pre-clinical and clinical studies on the protective effect of drinking raw milk on allergic disease [342,343,344]. The allergy-protective effect of raw milk was associated with epigenetic changes in mice [392]. Whether these protective effects are mediated by miRNAs or an effect of other bioactive components needs to be investigated in future studies.

### 5.5. Lactoferrin

Lactoferrin is a major protein of human milk, with the highest levels in colostrum. It is relatively stable in the gut because of its stability to enzymatic degradation. In addition to lactoferrin’s antimicrobial properties, there are also indications of its immunomodulatory effects in allergic disease [373,393]. As an example, it inhibits the production of pro-inflammatory cytokines such as TNF-α and IL-1β by binding to pathogen-associated molecular patterns (PAMPs) like lipopolysaccharide (LPS) and CpG-containing DNA. The binding to CpG-containing DNA led to the hypotheses that lactoferrin can act as a pre-transcriptional factor [376,394] via the regulation of NF-κB activation in the gut.

### 5.6. Vitamin D

Vitamin D is a fat-soluble prohormone obtained through sunlight or via nutrition, but a large number of women and children fail to meet the 400–600 international units of vitamin D. A relatively high rate of vitamin D deficiency is observed in woman and infants [395]. Maternal vitamin D supplementation during pregnancy and lactation has been shown to affect the risk of eczema in the offspring [359], although results are inconclusive [360]. Vitamin D is known to regulate DNA methylation and Anderson et al. showed that maternal vitamin D supplementation alters DNA methylation of genes related to developmental processes in breastfed infants [218]. A loss of methylation was associated with genes which play a role in metabolic processes and signal transduction pathways, also observed in the leukocytes of their lactating mothers [218]. Whether these epigenetic changes resulted in less atopic manifestations was not investigated in this study.

### 5.7. Human Milk Oligosaccharides (HMO) and SCFA

The HMO in breast milk constitute about 20% of the milk saccharides next to the major carbohydrate in milk, lactose. Human breast milk contains approximately 5–15 mg/mL of these non-digestible HMO, consisting of up to 200 or more unique structures. HMO are the key factor shaping the development of the immunity and early microbiota after birth. Colonization of the intestine begins during birth and commensal bacteria play a key role in programming the neonatal immune system [387,396], and infants who ultimately develop allergic disease harbor a distinct intestinal microbiome [397]. Gut microbial alterations are not limited to shifts in the abundance of certain microbes; they also include alterations in microbiota metabolism and changes in the production of microbial-derived metabolites such as SCFA [398]. Dietary factors can influence epigenetic regulation of gene expression through an indirect mechanism mediated by modulation of gut microbiota. The major metabolites produced by gut microbiota are SCFA, such as butyrate, which have multiple beneficial effects at the intestinal and extra-intestinal level. As more dietary fibers are ingested, SCFA production increases. Several studies have implicated butyrate, propionate, and acetate as potent modulators of the mucosal immune system and SCFA are considered epigenetic modifiers of early life immunity, especially in relation to asthma development [399,400]. In relation to food allergy, it has been shown that butyrate induced *FOXP3* demethylation in peripheral blood mononuclear cells from children affected by challenge-proven IgE-mediated cow’s milk allergy [401]. Human milk collected from mothers 1 month postpartum contained detectable levels of the SCFA butyrate, acetate, and formate [402]. Interestingly, this study showed significantly lower concentrations of acetate and butyrate in breastmilk from atopic mothers compared to breastmilk from non-atopic mothers and depended on the country of residence [402]. These data implicate that reduced exposure to SCFA, directly or indirectly via the fermentation of HMO by the gut microbiome, may program allergic disease. The epigenetic effects of SCFA might underlie these observations.

In Chapter 5, we described the epigenetic mechanisms of bioactive human milk components with a protective effect in allergic disease.

## 6. Dietary (mi)RNAs and Allergy

In addition to the major energy-providing components, i.e., proteins, sugars, and lipids, natural foods contain large amounts of nucleic acids of both types, RNA and DNA, at different structural levels from complex poly- and oligonucleotides to single nucleotides. Until recently, it was thought that the complex structures are completely digested to single molecular components such as nucleotides and nucleosides which then serve as building blocks for the generation of autologous oligonucleotides. Within the past years this concept has been scrutinized by diverse demonstrations of circulating oligonucleotides including RNA molecules of foreign origin which became feasible by the latest developments of highly sensitive and specific molecular biology methods.

Based on these observations, the term dietary RNAs was generated to describe multinucleotide RNA complexes that entered the body via the digestive tract as food components. In fact, the existence of such dietary RNAs has been controversially discussed over the last years because the stability of otherwise quite sensitive RNA molecules during intestinal passage was questioned. It was even argued that foreign RNA detected in sera might have originated from a cross-contamination with exogenous RNAs [403]. However, in the meantime some specific structural features of certain small RNAs that withstand adverse conditions in the gastrointestinal tract have been identified, such as 2′-O-methylation at 3′-end [404], high G/C content, and/or stem loop structures [405] of small non-coding (snc)RNAs including miRNAs. Further, with gaining interest in the functional role of sncRNAs in intercellular communication processes, it has become obvious that such RNAs may often become systemically distributed as cargo of EV structures such as exosomes. These RNA-containing vesicles may not only be important for communication within one organism, but also for cross-kingdom communication, a mechanism already known to be used by plants. For instance, certain plant parasites are able to silence genes from the host via sncRNAs to promote their invasion, as well is the host capable to suppress the infection by delivering sncRNAs to the parasites [406]. As a specific example for cross-kingdom communication, cells of the plant *Arabidopsis* can secrete exosome-like EVs containing sncRNAs that are passed on to fungi to inhibit their pathogenicity by gene silencing [407].

Of specific interest here are the beneficial effects of orally acquired plant-derived sncRNAs on the health of mammals. The exogenous application of plant miRNAs has been shown to inhibit the development of diseases such as viral infections and cancer in the host [408], e.g., the small RNA miR-2911 derived from honeysuckle showed antiviral effects in mouse models of influenza virus infection [409] and the oral application of plant-derived tumor-suppressor miRNAs could also reduce colon cancer in a respective mouse model [410]. Therefore, it is not surprising that dietary sncRNAs were demonstrated to exert effects on host immune functions such as reduction in pro- and anti-inflammatory cytokine levels, e.g., IL-6 and IL-10 [411,412]. In addition, oral uptake of yeast-derived RNA reduced systemic glucose levels in a high-fat diet model system in the presence of B and T lymphocytes [411].

Furthermore, tissue repair and renewal processes could be activated by grape exosome-like nanoparticles, which target stem cells [413], and orally-given ginger-derived nanoparticles were identified as a useful therapeutic tool with advantages compared to synthesized nanoparticles [414]. In addition, it is known that dietary RNAs from ginger can interact with gut microbiota, together leading to an improvement of the intestinal epithelial barrier function by increasing the local production of IL-22 [415]. In addition, miR-168a, present in plentiful amounts in rice and found in human plasma and organs after respective food intake, was shown in mice to upregulate low-density lipoprotein (LDL) plasma levels by binding to the *LDLRAP1* gene and thus inhibiting its expression in the liver [416]. Such mechanisms were not limited to plant sncRNAs, since a good bioavailability was also demonstrated for bovine miRNAs encapsulated in cow’s milk exosomes which were found in several tissues of mice after oral uptake [417]. Not only in mice, but also in adult humans subjected to dietary cow’s milk, increased plasma levels of miRNA-29b and miRNA-200c were found. The functional effects of such miRNAs were assessed in vitro by treating HEK cells with milk exosomes, resulting in a significant decrease of the respective reporter gene activity [418].

In conclusion, dietary RNAs from animal (interspecies transfer) or plant (cross-kingdom communication) sources may interact with the gut or the host’s microbiome or may even become systemically distributed. Such exogenous sncRNAs have been shown to affect gene expression in target cells and thus may affect cellular regulation processes of the host. By these mechanisms, this class of bioactive molecules may contribute to the impact of food on human health [419]. Especially in the perinatal and early postnatal period, such effects may participate in the generation of steady-state conditions essential to develop and maintain a healthy constitution on the one hand, while on the other hand, they may interfere with the development of and/or protection from several diseases, especially those with an inflammatory component. So far, no reports have shown a direct association of dietary sncRNAs with allergy outcomes; however, based on the current developments in this emerging field of nutrigenomics, it is more than likely that such observations will come up in the near future. In consequence, deeper insights into the role and underlying mechanisms of interspecies/interkingdom sncRNA transfer may lead to further optimization or development of completely new molecular nutrition concepts.

## 7. Immunometabolism in Allergy in Relation to Epigenetics

Immune cells, as all other cells, function properly depending on their cellular metabolic reprogramming. This term describes the complex interactions between cellular catabolic processes, producing energy in a form of adenosine triphosphate (ATP) and anabolic processes, using produced energy to build up necessary products. Catabolism includes glycolysis, oxidative phosphorylation, and FA oxidation, whereas anabolism defines processes of nucleotides, protein, and FA synthesis. Additionally, there are other intracellular pathways which play an important role in regulating cellular metabolism, such as hypoxia-inducible factor 1-α (HIF-1α), mammalian target of rapamycin (mTOR), and ubiquitination pathways. Metabolic reprogramming is involved in all kinds of cellular processes consisting of transcription, translation, nucleotide, protein, and FA synthesis, which are related with cellular functions, such as cellular activation, production of active mediators, proliferation, or migration. Several lines of evidence suggest that atypical metabolic reprogramming induced by extrinsic factors such as allergens, viruses, pollutants, diet, microbiome, as well as intrinsic factors such comorbidities, obesity, or stress might drive cellular dysfunctions and defective immune responses in allergic and other inflammatory diseases [420,421,422,423]. There is a tight link between metabolic reprogramming, epigenetic changes, and proper function of the immune cells and as such there is a high probability that these mechanisms are modulated already in utero and in early life, yet the evidence is scarce.

Modifications of metabolic reprogramming have been suggested to take place in innate and adaptive immunity, in the context of pathophysiology of allergic diseases (Table 3). It has been shown that metabolic processes are involved in the epithelial barrier impairment, proper functions of ILCs, mast cells, macrophages, and DCs. Epithelial HIF-1α/claudin-1 axis maintains the epithelial barrier function in eosinophilic esophagitis [424]. Autophagy is critical for FA oxidation and glycolysis in ILC2s, which is further associated with ILC2-mediated airway hyperresponsiveness [425]. IL-33 activates glycolysis with increased subsequent cytokine production in mast cells in allergic asthma. Cytokine production is suppressed by 2-deoxyglucose (2-DG) via blocking glycolysis, but could be rebounded while co-stimulated with ATP [426]. Regarding epigenetic changes in innate immune cells via metabolic reprogramming, α-ketoglutarate (αKG) generated from glutaminolysis plays the core role in epigenetic modulation of macrophage M2-like genes via jumonji domain-containing protein D3 (Jmjd3), a key enzyme for demethylation of H3K27, which is further related to the function of M2 macrophages [427]. Accumulation of mevalonate constitutes the specific epigenetic phenotype of myeloid cells by activating the insulin-like growth factor 1 (IGF-1) receptor (IGF1-R), mTOR, and histone modification [428]. Metabolic reprogramming and epigenetic changes play important roles in the innate cells’ memory, which is called “trained immunity” [429]. Trained immunity and tolerance have been recently shown to be induced in the ILCs and monocytes in allergic patients who undergo allergen-specific immunotherapy [430].

More is known about the importance of immune metabolism in steering adaptive immunity. Pyruvate dehydrogenase kinase (PDHK) or mevalonate synthesis results in reduced Th2 cells differentiation and decreased expression of Th2-related transcriptional factor, PPARγ [436]. Exosomes containing mitochondria exist in myeloid-derived regulatory cells in asthmatic subjects. They are internalized by CD4^+^ T cells and finally merge with the host mitochondrial network [437]. Ketogenesis-derived β-hydroxybutyrate epigenetically modifies histone H3 lysine 9 (H3K9) of murine *Foxo1* and *Ppargc1a* (encoding PPARγ coactivator 1-α–PGC-1α) via β-hydroxybutyrylation in CD8^+^ memory T cells [446]. Methionine is essential for maintaining intracellular methyl donor S-adenosyl-L-methionine (SAM) pools in T cells. Its restriction reduces methylation of histone 3 lysine 4 (H3K4) at the promoter regions of key genes involved in Th17 cell proliferation [447]. In addition, expression of the methyltransferase enhancer of zeste homolog 2 (EZH2) is induced by activation of spleen tyrosine kinase (Syk) and mTOR complex 1 (mTORC1) in the presence of methionine, leading to methylation of histone H3K27 at transcription factor BTB and CNC homolog 2 (Bach2), finally contributing to plasmablast differentiation [448].

Multiomic analysis increasingly gives us more insight into the field, e.g., transcriptomics has revealed that FA metabolism was significantly altered in severe allergic inflammation individuals, whereas metabolomic data suggested increased glycolysis in severe allergic patients, with higher levels of carbohydrates and pyruvate. In addition, severe allergic inflammation subjects showed downregulated histone modulation in transcriptomics [452]. Alterations in FA metabolism lead to increased production of active lipid mediators, such as eicosanoids (prostaglandins, leukotrienes, thromboxanes), other oxylipins, phospholipids, sphingolipids, and bile acids, which play a pleiotropic role in pathogenesis of allergy and asthma [128,449]. Abnormal FA metabolism is also involved in the pathogenesis of different asthma phenotypes and endotypes, and is also connected with viral exacerbations of asthma [450,451,455]. The existence of the gut–lung axis is partially explained by the effects of gut microbiota metabolites and their effects in the lung [456,457].

Taken together, metabolic reprogramming, including FA metabolism, glutaminolysis, ketogenesis, mitochondria merging, and others, plays a critical role in regulating epithelial barrier function, cytokine production, immune cell proliferation, differentiation, and other innate and adaptive immune responses in a variety of allergic diseases in relation to epigenetics. With its limited knowledge in utero and in early life, the near future should bring more data on the importance of the metabolic reprogramming during the perinatal period.

## 8. Conclusions

Epigenetic programming is key during development and the significant physiological changes occurring in the prenatal period make this timeframe starting from conception until the first years of life a critical window for epigenetic processes. Epigenetic modifications due to environmental triggers like pollution, antibiotics, and dietary malnutrition during this vulnerable period may therefore lead to lifelong phenotypic alterations and disease. It is generally accepted that environmental factors have a major contribution to the increased risk of developing allergic diseases. These epigenetic modifications are heritable, potentially reversible, and affect transcription processes (e.g., acetylation of methylation of histones or methylations of DNA itself) without affecting the DNA sequence.

The perinatal period has also gained enormous interest as part of the window of opportunity. In this review, we described the positive impact of nutrition during pregnancy and lactation on the epigenetic signature and its influence on the risk for developing allergic disease. We summarized the current knowledge on specific food components and the potential to decrease the risk of developing allergic diseases through epigenetic mechanisms.

## Figures and Tables

**Table 1:** Epigenetic effects of parasites and microbes or their metabolites on immunity.

Genus/ Species	Metabolic Product	Epigenetic Mechanism	Targeted Cells/ Cellular Target	Downstream Targets/ Effects	Human/ Murine	Prenatal/ Early Life	References
**Bacteria**							
*Acinetobacter lwoffii*	-	Histone acetylation	CD4^+^ T cells	Th1 and Th2 key genes	Murine	Prenatal	[240]
*Lactobacillus reuteri*	-	DNA methylation	CD4^+^ T cells	Multiple loci	Human	Both	[242]
Microbiota	Acetate	Inhibits histone deacetylases	Treg cells	Acetylation of the *Foxp3* promoter	Human and murine	Prenatal	[147]
	Butyrate	Inhibits histone deacetylases	Mast cells	↓*BTK*, ↓*SYK*, ↓*LAT*	Human and murine	No	[243]
			Gut	NF-κB	Human	No	[244]
**Viruses**							
RSV	-	↑ Histone demethylase	DCs	↑Th2 responses	Human and murine	(Early life)	[245]
RV	-	DNA methylation	PBMCs	*SMAD3*	Human	Early life	[246]
**Parasites**							
*Ascaris*	-	Histone acetylation	PBMCs	Type 2 immune genes	Human	No; infected adults	[247]
		DNA methylation	CD4^+^ T cells	Th1 inhibition	Human	No; aged 6 months–15 years	[248]
*Schistosoma*	-	DNA methylation	CD4^+^ T cells	Th1 inhibition	Human	No; aged 6 months–15 years	[248]
		Histone acetylation	Naïve T cells	↓histone acetylation at the of the *Il4* promoter; ↓Th2 differentiation	Murine	Prenatal	[249]
*Fasciola hepatica*	-	DNA methylation	Macrophages	↑anti-inflammatory properties	Murine	No	[250]
**Fungi**							
*Malassezia furfur*	-	Histone acetylation, histone methylation	Keratinocytes	Genes encoding IL-8 and β-defensin	Human	No	[251]

Th1/2 (cells), T helper type1/2 (cells); Treg (cells), regulatory T (cells); *Foxp3*, forkhead box P3 encoding gene; *BTK*, Bruton tyrosine kinase encoding gene; *SYK*, spleen-associated tyrosine kinase encoding gene; *LAT*, linker for activation of T cells encoding gene; NF-κB, nuclear factor κB; RSV, respiratory syncytial virus; DCs, dendritic cells; RV, rhinovirus; PBMCs, peripheral blood mononuclear cells; *SMAD3*, SMAD family member 3 gene; *Il4*, interleukin 4 gene; IL-8, interleukin 8.

**Table 2:** Association between bioactive human milk components and the development of allergic disease.

Milk Components	General Information	In Vitro Outcomes	In Vivo Outcomes	References
**Clinical studies**				
HMO		A large variation in the concentration of neutral oligosaccharides in individual colostrum samples	No association between 9 neutral oligosaccharides in HM colostrum and the risk of allergic disease up to age 18 months	[350]
	6’ sialyllactose, lacto-N-fucopentaose I, lacto-N-fucopentaose III, and disialyl-lacto-N-tetraose		↓ HMO in maternal milk associated with ↓ cow’s milk allergy in infants. A coexpressed cluster of HMO associated with an increased risk of CMA	[351]
	Child study		↑ fucosly-disialyl-lacto-N-hexaose, lacto-N-fucopentaose II, lacto-N-neotetraose, lacto-N-fucopentaose I, LSTc, fucosyllacto-N-hexose and ↓ lacto-N-hexaose, lacto-N-tetraose, 2′-fucosyllactose, disialyl-lacto-N-hexaose in HM ↓ risk of food sensitization at age 1	[352]
			FUT2-dependent HMOs ↓ risk of atopic eczema at age 2 in high-risk children. No effect 5 years	[353]
	Melbourne atopy cohort study		HMO profiles dependent (acid-Lewis vs. acid-predominant) association with allergic disease risks in childhood	[306]
	Systematic review		↓ LNFP-III in HM associated with protective effect against CMA	[354]
	Human monocytes	HMOs isolated from HM stimulate semi-maturation of human monocytes-derived DCs associated with ↑ IL-6, IL-10, and IL-20		[355]
TGFβ	Conflicting data on the role of TGFβ		↑ and ↓ TGFβ1 or TGFβ2 demonstrating ↓ allergy-related outcomes in infancy and early childhood. No strong association found by Khaleva and Ismail et al.	[335,356,357,358]
Vitamin D	Systematic review		↓ maternal vitamin D during pregnancy associated with ↑ risk of childhood eczema.Results are inconclusive	[359,360]
Polyunsaturated fatty acids	Systematic review		Insufficient evidence that HM polyunsaturated fatty acids influence the risk of childhood allergic diseases	[361,362,363]
Soluble CD14	Katsushika study		↓ sCD14 levels in HM associated with ↑ AD at 9 months. Other studies showed no effect or ↓ on eczema	[358,364,365]
sIGA	The PASTURE cohort study		↓ sIGA levels in HM associated with eczema and CMA	[339,366]
Cytokines	IL-1α, IL-6, IL-10		IL-1α, IL-6, IL-10 in HM associated with tolerance to CMA	[367]
	IL13		IL-13 in HM associated with higher risk of eczema	[368]
Lactoferrin	Lactoferrin-fortified formula milk		LF addition was associated with a significantly lower incidence of respiratory (wheezing) and diarrhea related illnesses	[369]
**Preclinical studies**				
Maternal IgG	Murine food allergy model		Human breast milk containing OVA-IgG allergen specific immune complex induced food tolerance	[346]
Butyrate	Murine model for food allergy in vivo, cell culture and PBMCs	Increased IL-10, IFN-γ and Foxp3 in PBMCs; increased defensin-3, mucus, and tight junction expression in enterocytes	Preventive effect of Butyrate (HM levels) on allergic symptoms in mice	[370]
Free amino acids	Murine food allergy model	Levels of glutamine and glutamate increase during first month of lactation	AA-fed mice showed lower acute allergic skin responses and prevented the whey-induced symptoms of anaphylaxis	[305,371]
Lactoferrin	Epithelial cell line	LF ↓ LPS-induced cellular inflammation		[372]
	Murine asthma model		LF ↓ pollen antigen-induced airway inflammation	[373]
Alkaline phosphatase Osteopontin	Murine food allergy model		ALP prior to sensitization ↓ reduced allergic symptoms	[345]
	Osteopontin-fortified formula milk		OPN in an infant formula (close to human milk levels) ↑ the proportion of circulating T cells	[374]
6’Sialyllactose, 2’ Fucosyllactose	Murine food allergy model		↓ symptoms of food allergy through induction of IL-10(+) T regulatory cells and stabilization of mast cells	[375]

HMO, human milk (HM) oligosaccharides; CMA, cow’s milk allergy; LSTc, sialyl-lacto-N-tetraose c; FUT2, fucosyltransferase 2; LNFP-III, lacto-*N*-fucopentaose LNFP III; DCs, dendritic cells; IL, interleukin; TGFβ, transforming growth factor beta; sCD14, soluble CD14; AD, atopic dermatitis; sIgA, soluble immunoglobulin A; LF, lactoferrin; IgG, immunoglobulin G; OVA, ovalbumin; PBMCs, peripheral blood mononuclear cells; IFN-γ, interferon γ; Foxp3, forkhead box P3; AA, amino acids; LPS, lipopolysaccharide; ALP, alkaline phosphatase; OPN, osteopontin.

**Table 3:** Immunometabolism in allergy in relation to epigenetics.

Main Findings Summary	References
Epithelial HIF-1α/claudin-1 axis maintains epithelial barrier function in eosinophilic esophagitis	[424]
Autophagy is critical for fatty acid oxidation and glycolysis in ILC2s which is associated with ILC2-mediated AHR	[425]
IL-33 activates glycolysis with increased subsequent cytokine production in mast cells in allergic asthma. Cytokine production is suppressed by 2-DG via blocking glycolysis, but could be rebounded while co-stimulated with ATP	[426]
Administration of GlcNAc changes glucose metabolism via HBP, leading to remarkable alleviation of systemic anaphylaxis and ear swelling induced by mast cell degranulation	[431]
GlcN is involved in HBP; the modification of glucose metabolism could explain its beneficial effect in treating atopic dermatitis. GlcN supplementation reduces allergic asthma and rhinitis via upregulating HBP pathway	[432,433]
E-NPP3, one of the nucleotide-converting ectoenzymes, hydrolyzes extracellular ATP on cell surface of basophiles and mast cells contributing to block of cell overactivation and reduced allergic inflammation	[434]
Pentameric procyanidin reduces glucose uptake and the production of L-lactate in activated CD4^+^ T cells. Apple procyanidins decrease the proliferation of splenic CD4^+^ T cells via interfering with glycolysis	[435]
PDHK or mevalonate synthesis, resulting in reduced Th2 cells differentiation, decreased expression of Th2-related transcriptional factor, PPARγ	[436]
Exosomes containing mitochondria exist in myeloid-derived regulator cells in asthmatic subjects. They are internalized by CD4^+^ T cells and finally merge with the host mitochondrial network	[437]
Immunometabolism contributes to limiting the access of transcription factors to the binding sites	[438]
ACL, converting glucose derived citrate to acetyl-CoA, is increasingly demanded in histone acetylation in response to growth factor stimulation	[439]
In IL-4 treated macrophage, Akt-mTORC1 pathway influences histone acetylation of a series of M2 genes through modulating acetyl-CoA biosynthesis which finally contribute to altered cell proliferation and chemokine productions	[440]
Allergen-specific immunotherapy induces trained immunity changes in the phenotype and repertoire of innate lymphoid cells and monocytes	[430]
αKG generated from glutaminolysis plays the core role in epigenetic modulation of M2 genes via Jmjd3, enzyme for demethylation of H3K27. Macrophage M2 activation also relied on αKG-Jmjd3 pathway functions	[427]
Accumulation of mevalonate constitutes specific epigenetic phenotype of myeloid cells by activating IGF1-R, mTOR and histone modification	[428]
Elevated glycolysis is found in trained monocytes with high glucose usage, lactate induction, and NAD+/NADH ratio. The metabolic change relies on dectin-1/Akt/HIF-1α pathway in the mTOR activation dependent manner	[441]
Fumarate is augmented in response to glutamine supplementation by TCA cycle, which further results in epigenetic reprogramming in monocytes via inhibiting KDM5 histone demethylase	[442]
Increased mitochondrial superoxide causes general disruption in T cell DNA methylation and hydroxymethylation	[443]
Malate-aspartate shuttle, mitochondrial citrate export, and complex I supply the substrates needed for proliferation and epigenetic remodeling early during T cell activation	[444]
Increased transamination leads to increased levels of 2-hydroxyglutarate in differentiating Th17 cells resulting in hypermethylation of the Foxp3 gene locus and inhibited Foxp3 transcription	[445]
Ketogenesis-derived β-hydroxybutyrate epigenetically modifies H3K9 of murine *Foxo1* and *Ppargc1a* encoding PGC-1α via β-hydroxybutyrylation in CD8^+^ memory T cells	[446]
Methionine is essential for maintaining intracellular methyl donor SAM pools in T cells. Its restriction reduced H3K4me3 at the promoter regions of key genes involved in Th17 cell proliferation	[447]
The expression of methyltransferase EZH2 is induced by activation of Syk and mTORC1 in the presence of methionine, leading to methylation of histone H3K27 (H3K27me) at transcription factor Bach2, finally contributing to plasmablast differentiation	[448]
Altered metabolism of fatty acids, sphingolipids, and eicosanoids in asthma	[128,449,450,451,452]
Arginine metabolism endotypes relates to asthma severity	[453]
Altered fatty acids, bile acids, amino acid metabolites in asthma	[454]

HIF-1α, hypoxia-inducible factor 1-α; ILC2s, type 2 innate lymphoid cells; AHR, airway hyperresponsiveness; 2-DG, 2-deoxyglucose; ATP, adenosine triphosphate; GlcNAc, acetylglucosamine; HBP, hexosamine biosynthetic pathway; GlcN, glucosamine; E-NPP3, ectonucleotide pyrophosphatase/phosphodiesterase 3; PDHK, pyruvate dehydrogenase kinase; Th2 (cells), T helper type 2 (cells); PPARγ, peroxisome proliferator-activated receptor-γ; ACL, ATP-citrate lyase; acetyl-CoA, acetyl coenzyme A; IL-4, interleukin 4; Akt, protein kinase B; mTORC1, mammalian target of rapamycin (mTOR) complex 1; αKG, α-ketoglutarate; H3K27, histone 3 lysine 27; Jmjd3, jumonji domain-containing protein D3; IGF1-R, insulin-like growth factor 1 receptor; NAD+/NADH, nicotinamide adenine dinucleotide /nicotinamide adenine dinucleotide reduced; TCS, tricarboxylic acid; KDM5, lysine demethylase 5; Th17 (cells), T helper type 17 (cells); Foxp3, forkhead box P3; H3K9, histone 3 lysine 9; PGC-1α, PPARγ coactivator 1-α; H3K4me3, trimethylation of histone 3 lysine 4; SAM, S-adenosyl-L-methionine; EZH2, enhancer of zeste homolog 2; Syk, spleen tyrosine kinase; Bach2, BTB and CNC homolog 2.

## Data Availability

The datasets generated during and analysed during the current study are available from the corresponding author on reasonable request.
